# Anthropogenic influence of temperature changes across East Asia using CMIP6 simulations

**DOI:** 10.1038/s41598-022-16110-9

**Published:** 2022-07-13

**Authors:** Shaik Allabakash, Sanghun Lim

**Affiliations:** 1 283, Korea Institute of Civil Engineering and Building Technology, Goyang-daero, Ilsanseo-gu, Goyang-si, Gyeonggi-do 10223 Republic of Korea; 2 Météo-France, Centre de Météorologie Radar, Toulouse, France

**Keywords:** Climate change, Attribution

## Abstract

The present study explores the impact of anthropogenic forcings (ANT) on surface air temperatures (SATs) across East Asia (EA) over a long period (1850–2014) using the new Coupled Model Intercomparison Project Phase 6 (CMIP6) datasets. Based on CMIP6 multi-model ensemble simulations, the historical simulations (twentieth century) and future (twenty-first century) SAT projections were investigated. Our calculations show that during 1850–2014, the combination of ANT and natural (NAT) (‘ALL = ANT + NAT’) forcings increased the EA’s SAT by 0.031 °C/decade, while a high increase of 0.08 °C/decade due to greenhouse gas (GHG) emissions. The ANT forcing rapidly increased after 1969. As a result, SAT change was enhanced at a rate of 0.268 °C/decade and 0.255 °C/decade due to GHG and ALL forcings, respectively. Human-induced GHG emissions were the dominant factors driving SAT warming and will also contribute to substantial future warming trends. Additionally, the optimal fingerprinting method was used to signify the influence of ANT forcing on climate change in EA. In a two-signal analysis, the ANT forcing was distinctly detected and detached from NAT forcing. In three-signal analyses, GHG forcing was dominant and separated from AER and NAT forcings. The future projections from 2015 to 2100 were examined based on CMIP6 socioeconomic pathway emission scenarios.

## Introduction

The world faces the major challenge of ongoing climate change, which greatly impacts socioeconomic and human activities as well as human health^1^. Climate change has greatly increased the risks of biodiversity loss, as well as ecosystem degradation, damage, and transformation. It is also responsible for extreme weather events and slow onset, which has a negative impact on the economy^1^. According to IPCC 2022^1^, the surface air temperature (SAT) increased by 1.09 °C (0.95–1.20 °C) in the last decade (2011–2020), which is higher than the increase in 1850–1900. IPCC 2022^1^ also predicted that warming would exceed 1.5 °C in the near future. Many authors have investigated the effects of external forcing on mean temperatures at global and regional scales, with increases primarily attributed to anthropogenic greenhouse gas (GHG) emissions alongside other human forcings^2–5^. Meehl et al.^6^ explored a combination of natural and anthropogenic forcing using a parallel climate model, finding that natural (solar) forcing dominated warming during the early twentieth century and anthropogenic forcing (GHG emissions) dominated the late 21st-century warming. Egorova et al.^7^ also reported an annual mean global warming rate of 0.3 K during the early twentieth century (1910–1940), with approximately half of this warming attributed to CO_2_, CH_4_, and N_2_O (well-mixed GHGs), and approximately one-third attributed to solar irradiance.

East Asia (EA) is one of the regions vulnerable to climate change^8–12^. It is one of the most densely populated regions, containing several major industrial and agricultural centers including high-altitude areas such as the Tibetan Plateau. The EA region is sensitive to climate change that impacts the global climate as well. Therefore, understanding and assessing climate change across EA is important for estimating and predicting global climate change^8^. Increasing SAT trends in EA have been linked to extreme weather events^9,10^ including heatwaves^11^ and intense precipitation^12^. In response to these challenges, many studies have described the characteristics of SAT changes in EA countries. In China, SAT was increased by 0.78 ± 0.27 °C^13^ with a warming of 0.25 °C/decade and 0.17 °C/decade between 1961 and 2005 as a result of GHG emissions and other anthropogenic factors, respectively^14^. The emission of GHGs was the dominant factor forcing impacting climate change in China^13,14^. Across the Korean Peninsula, extreme heatwave events have also been associated with climate change^15^. In North Korea, Om et al.^16^ observed a temperature change of 0.21 °C/decade from 1918 to 2015, which is higher than the changes reported for mainland China and the global average. Coastal areas were found to experience lower warming trends than inland regions in North Korea. Similarly, Jung et al.^17^ observed that mean annual SATs in South Korea increased at a rate of 0.23 °C/decade between 1954 and 1999, with a higher warming rate during winter. Chung and Yoon^18^ found that GHG emissions increased annual mean temperature by 0.42 °C/decade across the Korean Peninsula between 1974 and 1997, with larger cities experiencing faster warming trends than rural areas and small cities. Wang et al.^19^ observed 0.35 °C/decade warming in northeast China and a 0.2 °C/decade warming in Hokkaido, Japan, between 1951 and 2000. They also observed that extreme high- and low-temperature events were significantly positively correlated between these two regions and the warming recorded in these regions was in the context of global change.

Most of the studies mentioned above focused on climate change in China and few studies have focused on Korea or Japan. Furthermore, most studies have considered the period 1950–2010 and employed Coupled Model Intercomparison Project Phase 3 (CMIP3) and Phase 5 (CMIP5) model simulations. To the best of our knowledge, CMIP6 datasets have not yet been used to examine EA climate trends during the historical period from 1850 to 2014 nor have future projections (2015–2100) been provided at this scale. There are also no detection and attribution studies of climate change across the whole of EA. To address this gap, here we focus on SAT changes in EA between the second half of the nineteenth century and the twenty-first century based on the new state-of-the-art multi-model CMIP6 simulations. This allows us to describe the contributions of external and natural forcings on climate change in this region. Based on our analysis, we also describe the relative contributions of each forcing in each EA country as well as across the EA as a whole. The main focus of the study is to examine the effect of human-induced/anthropogenic forcings on climate change over EA using CMIP6 simulations. We also provide future projections for each EA country and the entire EA region based on three different scenarios. We have also described the temperature trends after 1970, which indicates the effect of industrial revolution over EA region during this period.

The rest of the paper is structured as follows. Discussion of our results including observed and simulated temperature trends in response to the different forcing factors, the detection and attribution results, the observation-constrained future projections and the discussion of our findings are presented in the following sections. The data and methodology are described in the last section.

## Results and discussion

### Observed and model simulation trends

**Figure 1:**
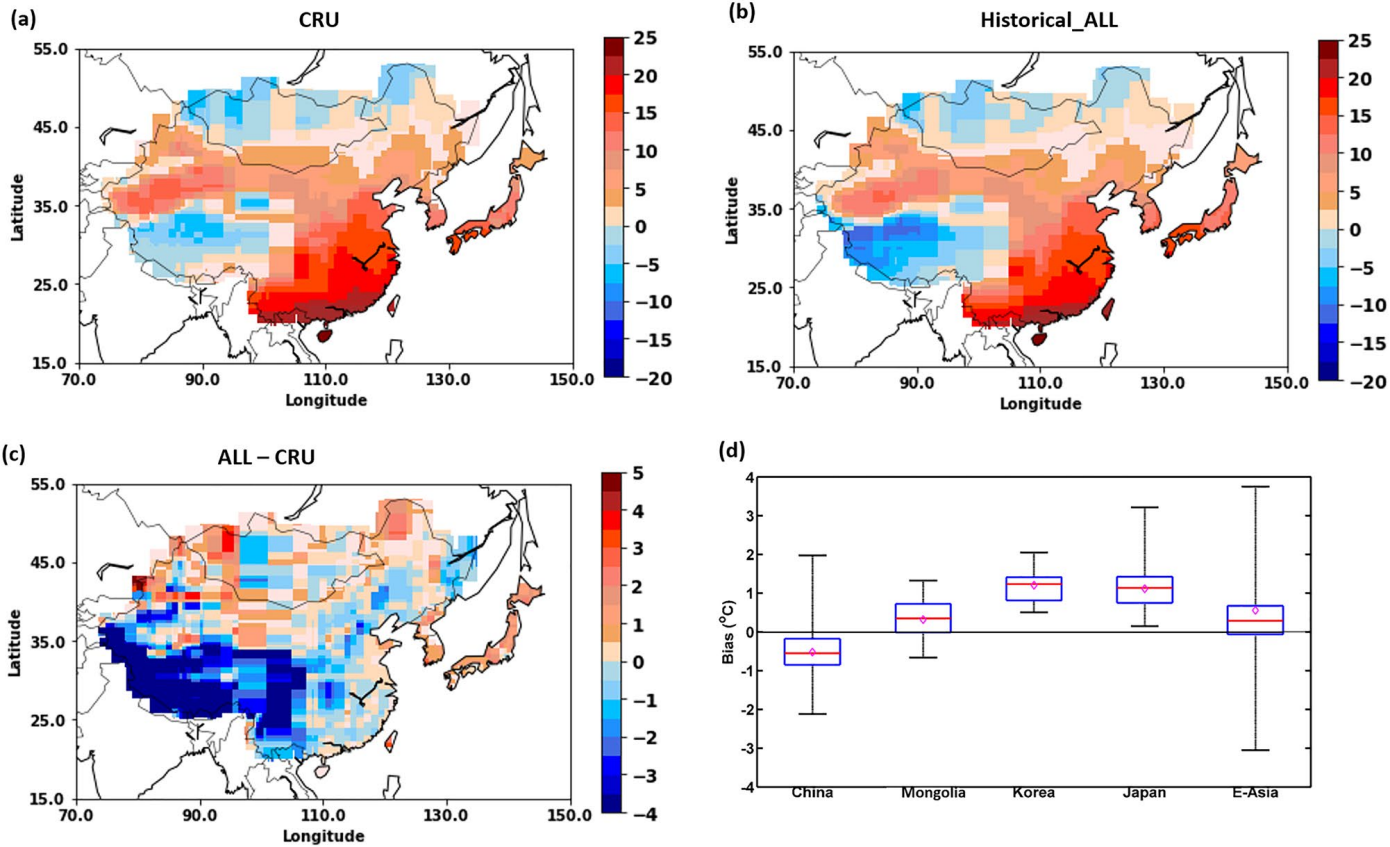
Annual mean temperatures from 1901 to 2014. (**a**) CRU, (**b**) multi-model ensemble mean of surface air temperature responses to ALL (anthropogenic and natural) forcing obtained from CMIP6. (**c**) Bias between ALL and CRU (ALL − CRU) across EA. (**d**) Bias (ALL − CRU) boxplots showing 5% (bottom of the whisker), 25% (bottom edge of the box), mean (diamond), median (central red line), 75% (top edge of the box), and 95% (top of the whisker) values. All figures were generated using licensed MATLAB (release R2017b available at https://in.mathworks.com/products/matlab.html).

Figure 1a, b show the annual mean temperatures averaged for 1901–2014 estimated from Climatic Research Unit (CRU) and CMIP6 historical simulation (ALL forcings = anthropogenic + natural), respectively. The mean temperature varied between − 15 and 25 °C throughout EA. Southeast China showed the highest mean temperature (approximately 25 °C), while Tibet and northwest Mongolia showed the lowest (< 0 °C). In Korea and Japan, the mean temperature varied from 4 to 16 °C. The performance of the CMIP6 model simulations (ALL forcing) was validated in comparison with the CRU observations. The bias (ALL − CRU) across EA and in all EA countries is shown in Fig. 1c, d. In Fig. 1d, the 25% and 75% quartiles indicate a bias of < 1.8 °C. Overall, the mean bias for EA was ≤ 0.5 °C, indicating that the performance of the CMIP6 simulations was satisfactory. Figures 1a, b also show that the mean temperature values of CRU and CMIP6 (ALL forcing) outputs are consistent. The Student’s t-test also showed no significant differences between the observations and model estimations at the selected time period (significance level < 0.05). In addition, we also evaluated the spatial SAT trends for both CMIP6 (ALL forcing) and CRU datasets; these are shown in Fig. S1. The CMIP6 trends more closely match the CRU trends with a bias of less than ± 1. These trends show that northern EA experienced the highest warming trend, while southeastern China showed the lowest warming for the 1901–2014 period.

**Figure 2:**
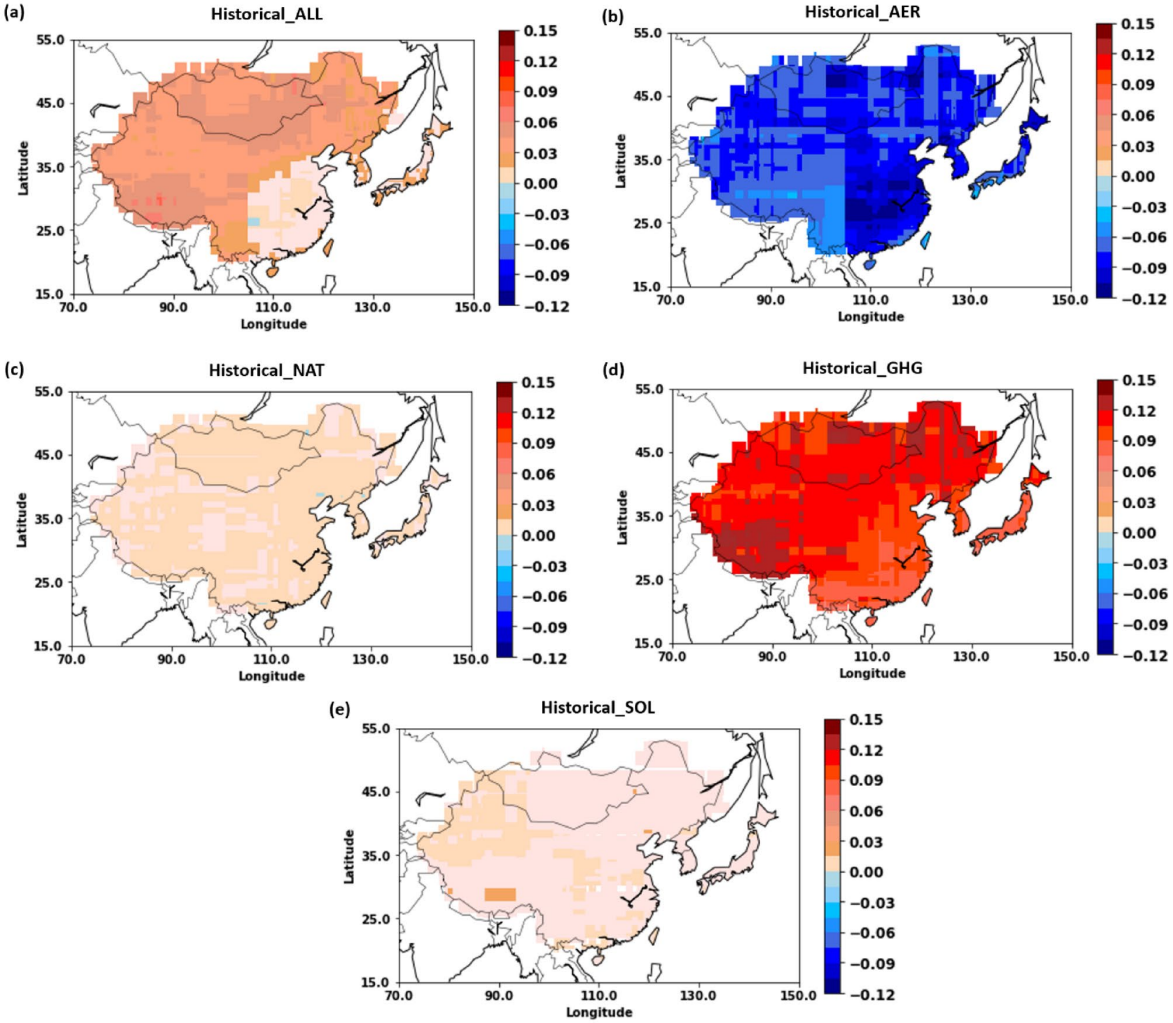
Spatial distribution of trends in annual mean surface air temperature responses to different forcings (°C/decade) for the historical period 1850–2014 obtained from CMIP6. (**a**) ALL, (**b**) anthropogenic aerosol (AER), (**c**) natural (NAT), (**d**) greenhouse gas (GHG), and (**e**) solar irradiance (SOL). The trends are estimated based on robust regression analysis. All figures were generated using licensed MATLAB (release R2017b available at https://in.mathworks.com/products/matlab.html).

We calculated the spatial distributions of the annual mean SAT MME mean response trends (based on CMIP6) to the different forcings for the period 1850–2014. Figure 2a–e correspond to the temperature trends associated with the simulations of historical (ALL), hist-aer (AER), hist-nat (NAT), hist-GHG (GHG), and hist-sol (SOL) forcings. The trends are estimated based on robust regression fitting analysis. The lowest increases in temperature owing to ALL and GHG occurred in southeast China, while the highest increases were observed in Tibet and southern Mongolia. As the third industrial revolution began in 1969, we also estimated the temperature changes over the last 40 years (1971–2014; Table S1) shown in Fig. S2. The SAT response to ALL forcings clearly shows a positive trend throughout EA during the study period, with an increase of approximately 0.031 °C/decade between 1850 and 2014 (Period 1 = P1) and 0.255 °C/decade between 1971 and 2014 (P2). Overall, Mongolia showed the highest rate of increase at 0.049 °C/decade in P1 and 0.379 °C/decade in P2, and Japan the lowest at 0.026 °C/decade in P1 and 0.25 °C/decade in P2. AER showed negative and lowest temperature trend in all the EA countries (Fig. 2b and S2b; Table S1). Thus, it is determined that AER cools SAT and slows the warming rate. Across EA, the overall AER cooling trend was − 0.076 °C/decade during P1 and − 0.035 °C/decade in P2. Interestingly, in P2, Mongolia showed a positive/increasing trend in association with the highest degree of warming observed in this region. Figure 2c shows that the SAT values were less influenced by NAT forcing, and Table S1 shows approximately zero NAT values for P1 in all the EA countries compared to positive trends during P2. Consequently, SATs in EA remained relatively stable during P1 (0.008 °C/decade) and P2 (0.019 °C/decade) in response to NAT forcing. A massive volcano eruption was occurred on June 15, 1991, over Mount Pinatubo^20^; it is known that the explosive volcanic eruptions emit sulfur dioxide gas to the stratosphere, which is oxidized to sulfate aerosols within a few weeks. In the stratosphere, sulfate aerosols scatter incoming solar radiation, reducing the energy inflow on the earth’s surface. As a result, the surface temperature decreases for 2–3 years. There was no big volcano explosion after the Pinatubo volcano, which was the largest volcanic eruption since 1960. In the period 1971–2014, NAT models show a modest warming trend, which is most likely related to volcanic eruption recovery. The third industrial revolution (1969–2000) was most prominent in the region of China, Japan, and Korea, where significant development took place (for example, new industries and technologies were established)^21–23^. Thus, the surface temperature increased significantly over EA and accordingly global SAT was also increased (figure not shown). Tett et al.^24^ also found that temperatures increased by 0.1 °C from 1900 to 1960 and then subsequently (1960–1997) increased by 0.5 °C, owing to anthropogenic effects on a global scale. GHG forcing produced the warmest trend among all the forcings (Fig. 2d) corresponding to 0.082 °C/decade across all of EA during P1 and 0.268 °C/decade during P2. Mongolia, China, Korea, and Japan showed higher warming rates as a result of GHG forcing during P2 than during P1 (Table S1). A significant increase in GHG forcing effects can be seen for P2, again indicating the impact of industrialization during this period. The SOL forcing effects (Fig. 2e) show mostly stable trends during P1 and P2, with values of 0.005 and 0.008 °C/decade for the entire EA region, respectively (Table S1).

**Figure 3:**
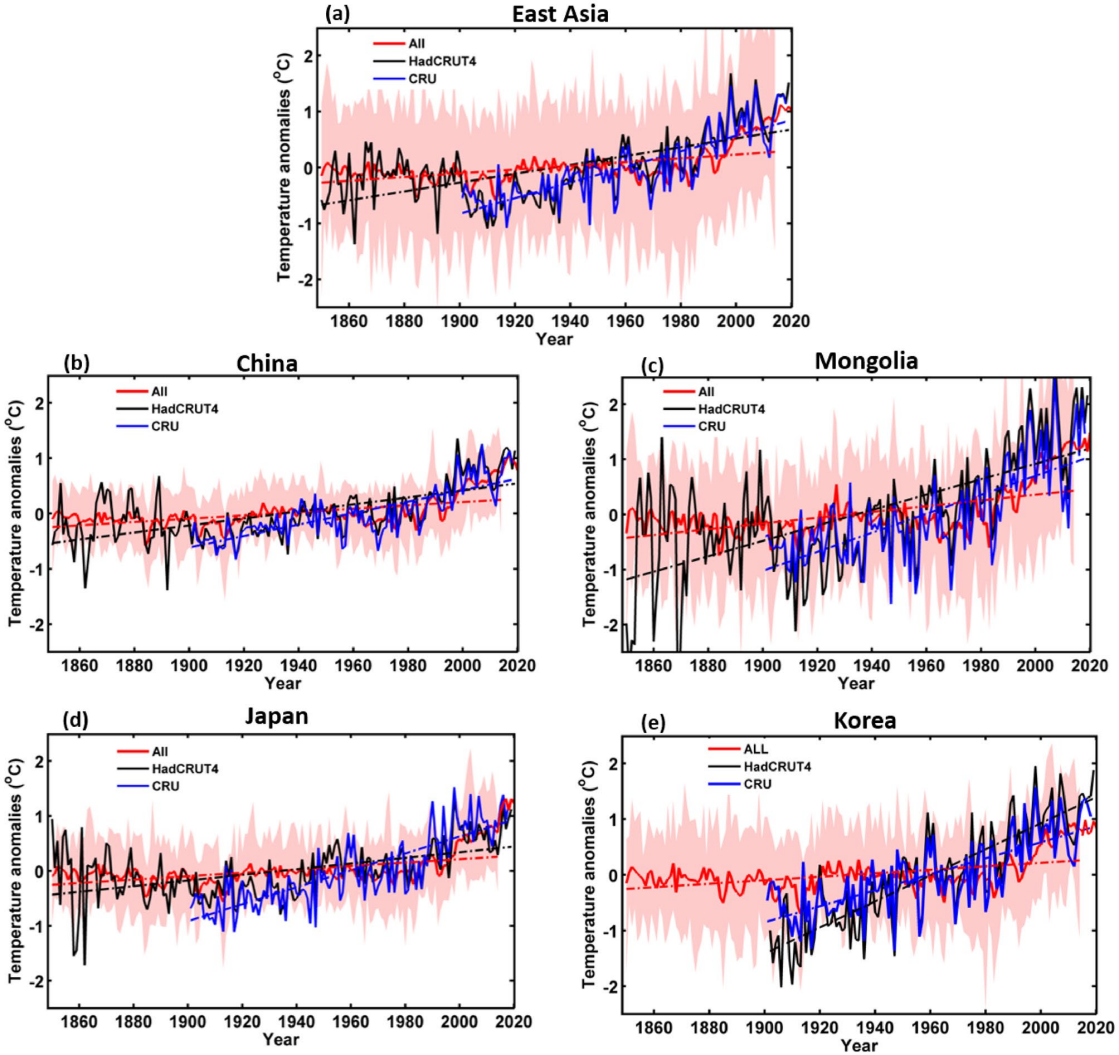
Temporal variation of annual mean temperature anomaly responses to ALL averaged across East Asia and individual countries from observations (CRU and HadCRUT4) and multi-model mean simulations (CMIP6) for the period 1850–2014 (CRU: 1901–2018, HadCRUT4: 1850–2019). Shaded bands are multi-model ranges.

**Figure 4:**
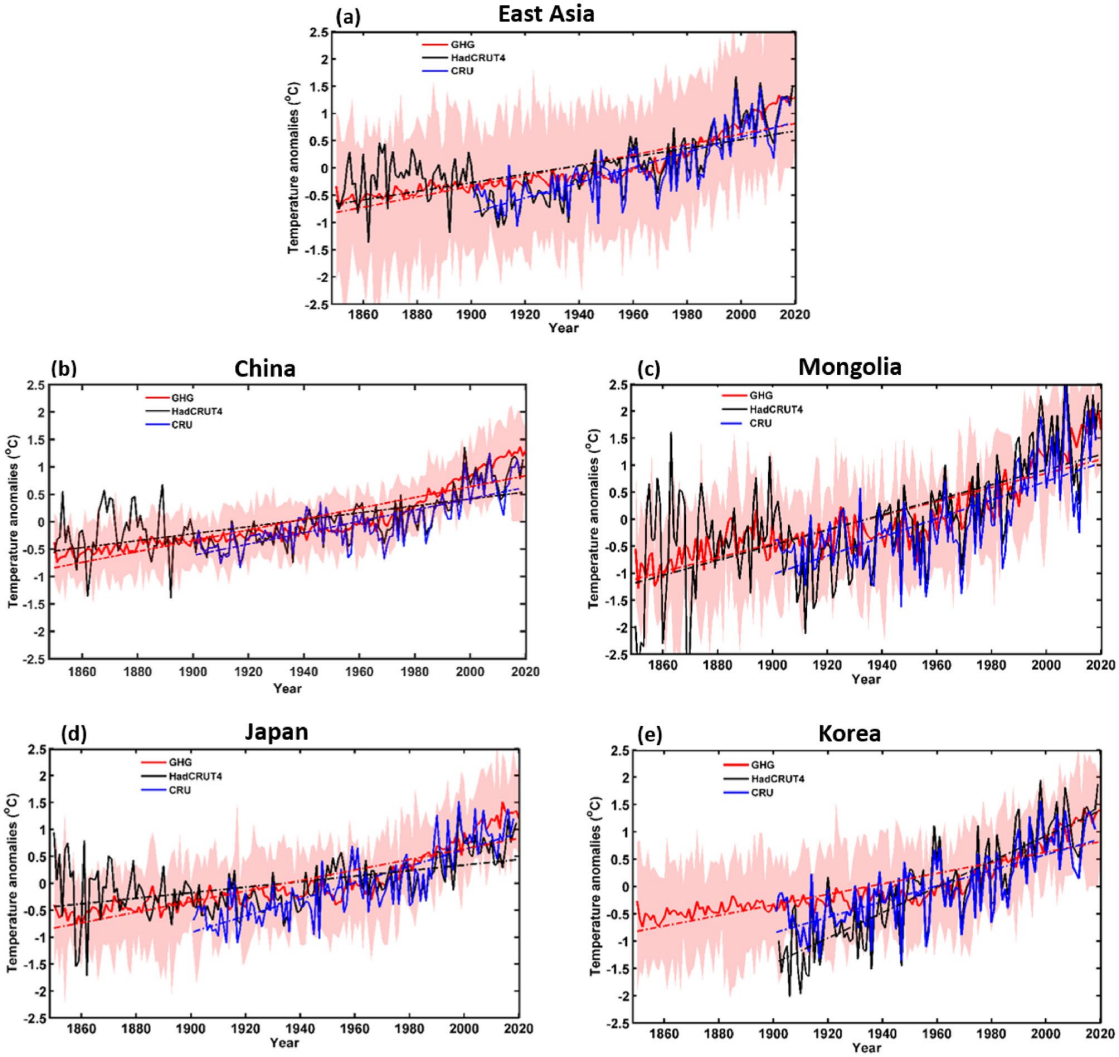
As for Fig. 3 but showing temperature anomaly responses to GHG forcing.

**Figure 5:**
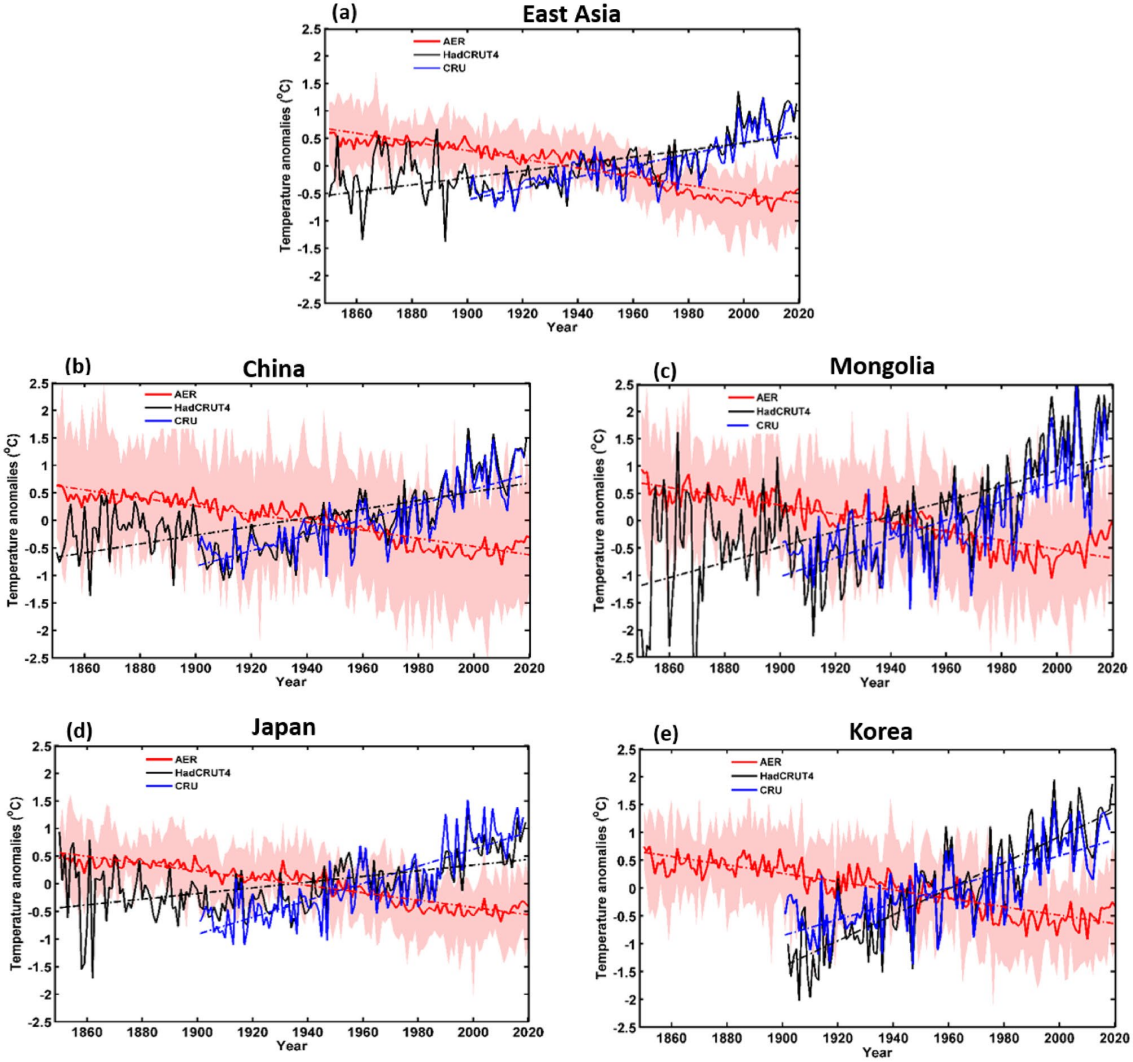
As for Fig. 3 but showing temperature anomaly responses to aerosol (AER) forcing.

The MME mean responses of ALL, GHG, AER, NAT, and SOL of each forcing were averaged for each EA country as well as for the whole of EA (Table 1 shows the models used for each forcing). The annual temperature anomalies for each forcing were calculated relative to the 1850–2019 period; anomalies were also estimated between 1850 and 2014, the period for which ALL forcing simulations were available. However, we used SSP2–4.5 scenarios from 2015 to 2019 to match the duration of ALL forcing simulations to the other forcing simulations. Figure 3 shows the temperature anomaly response to ALL forcing as well as ground observations, CRU (1901–2018), and HadCRUT4 (1850–2019) for all of EA and each country. Based on these results, the SAT trends associated with ALL forcings were consistent with the observed trends. The SATs in the HadCRUT4 and ALL datasets were mostly stable between 1850 and 1900, after which SAT dropped. This may have been caused by the cooling effect of AER. From 1901 to 1970, the observations (CRU and HadCRUT4) and the ALL data anomalies show slow increasing SAT trends. However, from 1970 to 2014/2019, SAT increased rapidly owing to the third industrial revolution. Across all of EA, SAT had increased by 1.5 °C by 2019, with trends in each country being broadly similar. Specifically, under the ALL forcings, all the East Asian countries experienced smaller increments of change between 1900 and 1969 compared to a higher rate of increase between 1970 and 2014. By 2019, the SATs in China and Japan had increased by ≥ 1 °C, while in Korea and Mongolia, the increase had reached ≥ 1.5 °C. The SAT anomaly response to GHG forcing is shown in Fig. 4 for the period 1850–2019, with observed and simulated SAT datasets showing a similar increasing trend. A slow increase in GHG forcing is observed until 1969, after which SATs begin to increase very rapidly. Indeed, GHG forcing has been the dominant contributor to the observed SAT warming in each country as well as across the whole of EA. For example, GHG forcing increased SATs by approximately 1.9 °C throughout EA and China by 2019, with even higher degrees of warming (≥ 2 °C) in Mongolia and Korea. In Japan, the equivalent temperature increase was ≤ 2 °C. The temperature trend response to AER forcing was negative throughout EA and in each country (Fig. 5), indicating that the cooling effect of AER forcing partially counteracts the warming caused by other forcings. The temperature trends in response to the AER were high over Mongolia followed by Korea and lower in Japan. At 1850, temperature trends were 1 °C over Mongolia, 0.78 °C in Korea, 0.58 °C over China and EA, and 0.51 °C over Japan. The decreasing trend of the temperature (1850–2019) was 0.98 °C over Mongolia and EA, and 0.9 °C over the other countries. Decreasing trend was deep from 1950 to 2000 compared to 1850 to 1950. The SAT changes in response to the NAT and SOL forcings are shown in Figs. S3 and S4, respectively, which remained broadly stable in all the EA regions indicating minimal forcing effects. The SAT changes in response to the NAT forcing were slightly high (0.24 °C) in Mongolia compared to other countries at 1850. However, the trend was mostly stable (≤ 0.15 °C) for EA and other countries while Mongolia with a small decreasing trend ≤ 0.3 °C. The mean trend of the NAT as well as SOL forcings was almost zero over all the EA countries as well as EA region. Figures 2, 3, 4 and 5 and Figs. S3–S4 illustrate the SAT response to ALL and GHG show positively correlated with the CRU SAT data. In contrast, AER is negatively correlated with the CRU data, and NAT and SOL show very weak/no correlation. It clearly demonstrates the direction (i.e., warming or cooling) and relative strength of the contributions of the different forcing factors to SAT changes over the entire study period. Specifically, ALL and GHG forcings are associated with SAT increases; AER forcing produced a cooling effect; and NAT and SOL forcings had very weak or no impact on the warming rate.

**Table 1:** Models used in the study for historical simulations (Y = ‘yes’ and ‘N’ = no). The numbers in parenthesis represent the ensemble sizes of the corresponding models.

Model name	Historical forcing (1850–2014)				
	ALL	AER	NAT	GHG	SOL
CNRM-CM6-1-HR	Y (1)	N	N	N	N
CESM2-WACCM	Y (4)	N	N	Y (3)	N
E3SM-1–0	Y (5)	N	N	N	N
BCC-CSM2-MR	Y (3)	Y (3)	Y (3)	Y (3)	N
MRI-ESM2-0	Y (5)	Y (5)	Y (5)	Y (5)	Y (4)
CIESM	Y (3)	N	N	N	N
CAMS-CSM1-0	Y (3)	N	N	N	N
INM-CM4-8	Y (1)	N	N	N	N
FIO-ESM-2–0	Y (3)	N	N	N	N
CMCC-CM2	Y (3)	N	N	N	N
HadGEM3-GC31-MM	Y (6)	N	N	N	N
MIROC6	Y (9)	Y (4)	Y (7)	Y (3)	Y (3)
GISS-E2-1-G	Y (8)	Y (6)	Y (5)	Y (5)	Y (6)
CNRM-CM6-1	Y (6)	N	Y (4)	Y (9)	N
IPSL-CM6A-LR	Y (4)	Y (9)	Y (9)	Y (4)	N
CNRM-ESM2-1	Y (3)	Y (2)	N	N	N
ACCESS-CM2	Y (3)	N	N	N	N
ACCESS-ESM1-5	Y (6)	Y (3)	Y (3)	Y (3)	N
HadGEM3-GC31-LL	Y (4)	Y (4)	Y (4)	Y (4)	N
UKESM1-0-LL	Y (6)	N	N	N	N
CanESM5	Y (5)	Y (9)	Y (5)	Y (6)	Y (9)
GFDL-ESM4	N	Y (1)	Y (3)	Y (1)	N
CMCC-ESM2	N	N	N	N	N
MPI-ESM1-2-LR	N	N	N	N	N
CESM2	N	N	N	N	N
FGOALS-f3-L	N	N	N	N	N
NESM3	N	N	N	N	N
KACE-1–0-G	N	N	N	N	N
Total	21 (91)	10 (46)	10 (48)	11 (46)	4 (22)

### Detection and attribution analysis

**Figure 6:**
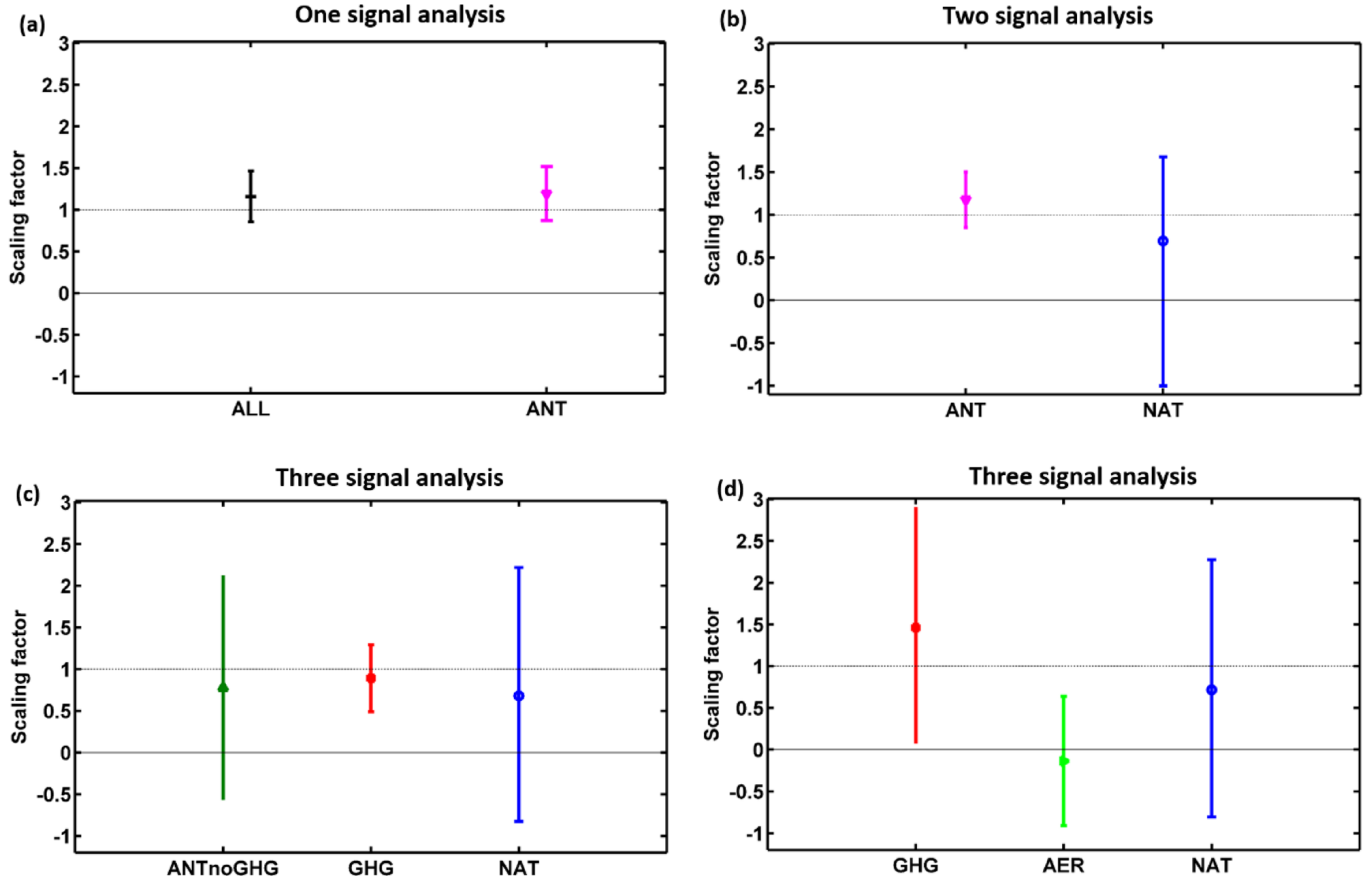
Best estimates of the scaling factors using the regularized optimal fingerprinting method with 90% confidence intervals over EA for the period 1905–2014. (**a**) One-signal analysis of all (ALL) and anthropogenic aerosols (ANT) forcings, (**b**) two-signal analysis of ANT and NAT forcings, (**c**) three-signal analysis of ANTnoGHG, GHG, and NAT, and (**d**) three-signal analysis of GHG, AER, and NAT.

To reduce sampling uncertainty in the detection and attribution analyses, we utilized an equal number of models and ensembles for each individual forcing (see Table S2). We conducted detection and attribution analysis based on the Regularized optimal fingerprinting (ROF) method for the annual mean temperature in EA over the last 110 years (1905–2014). The scaling factors, with 90% confidence intervals, of the one-, two-, and three-signal analyses are presented in Fig. 6. One-signal analyses were performed on the individual forcings of ALL (best estimate 1.15 with 90% confidence interval of 0.85 to 1.46) and anthropogenic (ANT) (best estimate 1.19 with 90% confidence interval of 0.87 to 1.51), as shown in Fig. 6a. The 90% confidence intervals of ALL, and ANT are above zero, indicating that these forcings are robustly detected in EA. The best estimate of ALL is greater than unity indicates the observed changes were underestimated by MME mean in response to ALL. Furthermore, the best estimates of ALL, and ANT are close to unity, indicating detection results pass the residual consistency test^25^, which representing the good agreement between model simulations and observed changes. The one signal analysis for all the EA countries is shown in Table 2, which was also mostly similar to the detection results of the EA region. The two-signal analyses were performed on the ANT (best estimate 1.18 with 90% confidence interval of 0.85 to 1.49) and NAT (best estimate 0.97 with 90% confidence interval of − 0.99 to 1.67) forcings, and the scaling factors are shown in Fig. 6b with 90% confidence intervals. The best estimates of ANT (1.18) and NAT (0.97) suggest that the effect of ANT can be separated from NAT. Further, ANT is robustly detected in EA, with a 90% confidence interval above zero; the best estimate is close to unity and is also comparable with ANT in the one-signal analysis, implying the robustness of anthropogenic influence on the observed temperature changes. In comparison, the lower bound of the NAT forcing is below zero, indicating that this forcing is undetected in EA. Therefore, only anthropogenic forcing can explain the observed annual mean temperature changes in EA from 1905 to 2014. Table 2 shows the ANT and NAT analysis for all the EA countries.

**Table 2:** Best-estimate scaling factors and 90% confidence intervals (shown in parenthesis) of different East Asian countries for the period 1905–2014.

1905–2014	East Asia (90%)	China (90%)	Mongolia (90%)	Japan (90%)	Korea (90%)
**ALL (anthropogenic and natural) and ANT forcings, estimated from one-signal analysis**					
ALL	1.15 (0.85–1.46)	1.14 (0.87–1.42)	1.17 (0.95–1.39)	1.21 (0.99–1.61)	1.22 (0.97–1.56)
ANT	1.19 (0.87–1.51)	1.18 (0.89–1.46)	1.26 (1.01–1.45)	1.31 (0.95–1.69)	1.23 (0.84–1.58)
**ANT and NAT forcings, estimated from two–signal analysis**					
ANT	1.18 (0.85–1.49)	1.2 (0.91–1.58)	1.19 (0.90–1.56)	1.28 (0.9–1.82)	1.27 (0.91–1.59)
NAT	0.97 (− 0.99–1.67)	0.39 (− 0.72–1.71)	− 1.36 (− 2.96–0.74)	1.05 (− 0.41–2.61)	0.58 (− 1.08–2.35)
**ANTnoGHG, GHG, and NAT forcing, estimated from three–signal analysis**					
ANTnoGHG	0.80 (− 0.50–2.05)	0.61 (− 0.35–1.56)	0.04 (− 1.15–1.19)	− 0.74 (− 2.11–0.45)	− 0.21 (− 2.18–0.54)
GHG	0.90 (0.48–1.31)	1.0 (0.57–1.42)	1.09 (0.81–1.35)	0.93 (0.51–1.33)	0.69 (0.16–1.18)
NAT	0.71 (− 0.80–2.24)	0.82 (− 0.91–2.68)	− 1.13 (− 3.64–1.21)	1.12 (− 0.19–2.71)	1.1 (− 0.6–2.95)
**GHG, AER, and NAT forcing, estimated from three-signal analysis**					
GHG	1.42 (0.09–2.72)	1.18 (0.06–3.01)	2.01 (0.04–1.59)	1.24 (0.09–3.14)	1.23 (0.08–3.12)
AER	− 0.13 (− 0.91–0.63)	0.18 (− 0.45–0.81)	− 0.09 (− 1.16–0.98)	− 0.58 (− 1.37–0.21)	− 0.42 (− 1.42–0.56)
NAT	0.71 (− 0.80–2.27)	1.04 (− 0.44–2.57)	− 1.03 (− 3.74–1.52)	1.29 (− 0.01–2.62)	0.92 (− 0.62–3.43)

We conducted the three-signal analyses using GHG (best estimate 0.90 with 90% confidence interval of 0.48 to 1.31), ANTnoGHG i.e., ANT–GHG (best estimate 0.80 with 90% confidence interval of − 0.50 to 2.05), and NAT (best estimate 0.71 with 90% confidence interval of − 0.80 to 2.24) to determine the major contributors among the anthropogenic forcings or other factors causing changes in the observations. This analysis also explored the influence of GHG forcing on SAT variations. Figure 6c shows the scaling factors for GHG, ANTnoGHG, and NAT. GHG is robustly detected in EA, with a 90% confidence interval above zero and the best estimate close to unity, which implies good agreement with the observations. In contrast, the lower bounds of the ANTnoGHG and NAT forcing include zero indicate that these are not detected in EA. Therefore, GHG forcing can be separated from ANTnoGHG and NAT forcing, and is considered the dominant anthropogenic factor forcing for the observed temperature changes in the study region. The results of the three-signal analysis for all the countries are shown in Table 2. To determine the GHG influence on SAT changes over other natural forcings, we conducted another three-signal analysis using GHG (best estimate 1.42 with 90% confidence interval of 0.09 to 2.72), AER (best estimate − 0.13 with 90% confidence interval of − 0.91 to 0.63), and NAT (best estimate 0.71 with 90% confidence interval of − 0.80 to 2.27). Figure 6d shows the AER and NAT 90% confidence intervals, which contain zero, indicating that these forcings are not detected, whereas GHG is detected over EA (90% confidence interval is above zero). As a result, GHG has become the dominant forcing over EA, having decoupled from AER and NAT. Table 2 shows the scaling factors for the other countries.

### Observation-constrained future projections

The one-signal analysis shown in Fig. 6a demonstrates that the best estimate of ALL forcings is 1.15 (above 1), which seems to possess underestimated the observed temperature changes. This historical underestimation could continue in future projections, requiring appropriate adjustment/correction to ensure the accurate estimation of future scenarios. Therefore, the MME mean future projections under the low future shared socioeconomic pathway (SSP) SSP1–2.6 and SSP2–4.5, and high SSP5–8.5 scenarios were multiplied by the best estimate of the ALL forcing scaling factor obtained in the one-signal analysis. Figure S5a shows the resulting historical (1850–2014) and future scenarios (2015–2100) for EA. These observation-constrained future projections show higher warming rates than the raw simulations. The adjusted/best estimate of the future projections of SSP1–2.6, SSP2–4.5, and SSP5–8.5 show temperature increases of 2.15 °C (90% confidence interval: 0.15–4.21 °C), 2.43 °C (90% confidence interval: 0.96–3.93 °C), and 2.97 °C (90% confidence interval: 1.49–4.46 °C), respectively by 2050 (average from 2041 to 2060), compared to 1.68 °C, 1.98 °C, and 2.23 °C based on the unadjusted simulations; by 2070 (average from 2061 to 2080), the best estimate temperature increases under SSP1–2.6, SSP2–4.5, and SSP5–8.5 are 2.35 °C (90% confidence interval: 0.39–4.42 °C), 3.0 °C (90% confidence interval: 1.51–4.49 °C), and 4.39 °C (90% confidence interval: 2.95–5.83 °C), respectively, compared to 2.06 °C, 2.34 °C, and 2.99 °C based on the original simulations; and by 2090 (average from 2081 to 2100), the best estimates under the same three scenarios are 2.48 °C (90% confidence interval: 0.44–4.55 °C), 3.51 °C (90% confidence interval: 2.03–4.98 °C), and 6.11 °C (90% confidence interval: 4.71–7.50 °C), compared to 2.11 °C, 2.69 °C, and 4.69 °C based on the original simulations, respectively. We also estimated the best values for the ALL forcing scaling factors for each EA country and generated projections for each EA country. The best estimates for the ALL forcing scaling factors, with 90% confidence intervals, for each EA country are shown in Table 2. The country-scale future projections under each of the scenarios were adjusted based on their respective ALL forcing best estimates, as shown in Fig. S5b–e. The best estimates of the future projections of SSP1–2.6, SSP2–4.5, and SSP5–8.5, with 90% confidence intervals, for each EA country in 2050 (average from 2041 to 2060), 2070 (average from 2061 to 2080), and 2090 (average from 2081 to 2100) are shown in Table S3. The resulting observation-constrained future projections in all the EA countries show higher warming rates relative to the raw simulation data, and warming is projected to increase over time.

We used the best estimate of GHG obtained from three-signal analysis, which is presented in Fig. 6d, to observe the effect of GHG forcing on future projections. The best estimate of GHG is 1.42, indicating that the GHG forcing contribution is also underestimated in terms of observed temperature changes and will continue in future scenarios. As a result, future scenarios were adjusted by multiplying them by the best GHG estimate (1.42). The impact of GHG forcings on future projections for the EA region is shown in Fig. S6a. The future estimates with observation constraints are warmer than the raw simulations. For all EA countries, Fig. S6 depicts the GHG-based future original simulations and their corrected scenarios. In comparison to the original simulations, all of the countries' updated scenarios indicate an increase. Table S4 shows the best estimate and 90% confidence intervals of GHG-based corrected future projections for all EA countries. Furthermore, Figs. S5 and S6, and Tables S3 and S4 indicate that GHG-based future scenarios project dominant warming than that of ALL forcings. Over EA, the GHG based future scenarios of SSP1–2.6, SSP2–4.5, and SSP5–8.5 produce temperature increases of 3.21 °C, 3.85 °C, and 4.72 °C, compared to 2.15 °C, 2.43 °C, and 2.97 °C in ALL forcing based projections, respectively by 2050 (average from 2041 to 2060); by 2070 (average from 2061 to 2080), the GHG based best estimate temperature increases under SSP1–2.6, SSP2–4.5, and SSP5–8.5 are 3.46 °C, 4.58 °C, and 6.52 °C, respectively, compared to 2.35 °C, 3.0 °C, and 4.39 °C based on ALL forcing simulations; and by 2090 (average from 2081 to 2100), the GHG based best estimates under the same three scenarios are 3.63 °C, 5.22 °C, and 8.70 °C, compared to 2.48 °C, 3.51 °C, and 6.11 °C based on the ALL forcing simulations. Overall, from Figs. S5 and S6, and Tables S3 and S4, it is inferred that the GHG-based future projections produce high warming rate compared to the ALL forcing based scenarios.

## Discussion

This study describes long-term SAT changes in EA using the new state-of-the-art CMIP6 multi-model simulations. These model simulations were validated in comparison with CRU/HadCRUT4 observational measurements. The SAT variation responses to various anthropogenic and natural forcings were examined between 1850 and 2014/2019. Throughout EA, southeast China has experienced the highest mean temperatures (approximately 25 °C) compared to the lowest on the Tibetan Plateau (approximately − 12 °C). SATs during the study period were increased due to GHG forcings and decreased due to the AER forcing. In contrast, NAT and SOL had little impact on SAT changes. GHG forcing was the dominant factor in the observed temperature increase. Overall, the SAT in EA increased by 0.082 °C/decade in response to the GHG forcing, compared to 0.031 °C/decade under ALL forcings from 1850–2014. After the third industrial revolution, SATs increased very rapidly, by 0.268 and 0.255 °C/decade in response to the GHG and ALL forcings, respectively, between 1970 and 2014. By 2019, the GHG forcing had increased the SAT across the EA by approximately 1.9 °C, and all countries in EA had also experienced increasing SAT trends as a result of anthropogenic forcings.

Overall, Mongolia experienced faster rates of temperature rise than other EA countries; however, throughout EA, the highest and lowest amounts of warming occurred in Tibet and southeast China, respectively. The strongest cooling response to AER forcing occurred in southeast China, while across EA, the overall cooling rate associated with AER forcing was approximately − 0.076 °C/decade between 1850 and 2014. Interestingly, in Mongolia, AER showed a warming rather than cooling influence during the 1971–2014 period, which might partially explain the high degree of warming in this region. Across EA, NAT and SOL forcings had a minimal effect on temperature changes. Based on these observations, anthropogenic forcing has significantly influenced the climate of EA, associated with distinct warming trends. Furthermore, we present future SAT projections up to 2100 based on the low SSP1–2.5 and SSP2–4.5, and high SSP5–8.5, scenarios.

We applied the ROF detection and attribution technique to CMIP6 simulations to describe climate change in EA resulting from anthropogenic influences. The ALL and ANT forcings were robustly detected from the one-signal analyses for the period 1905–2014; in the two-signal analysis, ANT and NAT influences could be separated, and the ANT forcing was clearly detected as a factor including the increase in SATs; and in the three-signal analysis, GHG forcing was separated from ANTnoGHG and NAT forcings and was strongly detected, indicating that GHG forcing was the dominant factor driving climate change in EA. The GHG forcing was separated from the NAT and AER forcings and was dominant over other forcings. Finally, we generated adjusted/corrected future warming trends by multiplying the raw simulation data with the ALL forcing best estimates, which produced higher projected temperature values under the SSP1–2.5, SSP2–4.5, and SSP5–8.5 scenarios. Further, GHG influence on the future projections was also calculated and the future scenarios were corrected with the best estimate of GHG forcing.

The future warming rate over the EA region was predicted to increase significantly by observation-constrained future projections. Therefore, EA is highly vulnerable to climate change which causes extreme weather events, accelerates glacier melting, and has a severe impact on ecosystems and agricultural activities. We found that the future estimates based on GHGs were warmer than the scenarios based on ALL forcings. Overall, we conclude that the climate (SAT) changes observed in EA are the result of anthropogenic forcings, primarily GHG. This implies that significant efforts and adaptation policies are recommended to slow the rate of warming caused by anthropogenic forcings.

In this study, we did not consider the impact of land use, urbanization, or local aerosol emissions on temperature changes. We used global climate models, which may not provide all topographical/geographical information (such as complex terrains) and local aerosol emissions. Therefore, we propose that we can mitigate these issues by employing accurate regional models that provide LU and local emission effects. Furthermore, we used various models with different boundary and initial conditions, parameters, and resolutions (high/low) that may produce uncertainty in the observations; consequently, the ensemble mean produces little uncertainty in the results. As a result, models may not closely match CRU observations in some regions (e.g. complex terrain and island areas). Hence, the models for the analysis should be chosen with care. The methodology used in the current study for the observation constrained future scenarios may also provide little uncertainty in the future scenarios due to inconsistency between historical and future projections. However, it can be reduced by employing the best method (downscaling/harmonization) with good synchronization between historical and future projections. Thus, we take all of these limitations into account and will conduct a more comprehensive investigation into the causes of climate change in the EA region as a future study.

## Methods

### Data

For the long-term variability analysis of SAT, we used the CRU time series version 4.03 (resolution = 0.5° × 0.5°) and HadCRUT4 (resolution = 5° × 5°) observational datasets for the period 1901–2018 and 1850–2019, respectively; the HadCRUT4 dataset (available at https://crudata.uea.ac.uk/cru/data/temperature/) was primarily used to cover the period not considered by the CRU dataset (1850–1900). The CRU data are derived from the spatial interpolation of monthly climate anomalies of worldwide weather station observations^26–28^ and are available at https://crudata.uea.ac.uk/cru/data/hrg/. These CRU data have been used by many researchers studying long-term temperature and precipitation changes over the EA region^26,27,29,30^ and they correlate well with ground station datasets. The land and ocean data were separated, and the former was employed in this study. We used geographical data available at https://www.geodatasource.com/addon/country-borders to identify the country borders (EA, China, Mongolia, Japan, and Korea), and the data within those borders were used in this study.

The state-of-the-art global multimodel simulations of CMIP6^31^, supported by the World Climate Research Program, were used to estimate the influence of different external forcings and are available at https://esgf-node.llnl.gov/projects/cmip6/. Some of the CMIP6 models are more biased when compared to observational datasets^32,33^. It is known that the various models have different boundary and initial conditions, parameter values, and structural uncertainties in the model design. Weather changes or predictions are sensitive to small difference/variation in the initial conditions of the models. Further, some models may have higher resolutions and others have lower resolutions, and accordingly, inter-model variability exists in terms of the different forcings that influence the climatology of the atmosphere. Thus, we used the MME mean to mitigate the systematic errors that arise from the individual models. The MME combines the strengths of the different models and diminishes the internal variability or uncertainty of the different models. In the present study, we selected the model simulations which are a better match with the observations. The MME mean significantly reduces the uncertainty of the selected model simulations. Several researchers were used the MME technique to study climate change across the global as well as individual regions^25,34,35^. Annual or decadal average of MME mean temperatures were mostly insensitive to small variations in initial conditions of the different models. Thus, the direct comparison between MME mean simulations and CRU observations shows a high spatial correlation (Fig. 3) indicating that the MME is less sensitive to the inter-model variations. Therefore, we used MME mean values to mitigate any bias and obtain the most accurate values possible. The various CMIP6 models (Table 1) were re-gridded using bilinear interpolation to achieve a uniform resolution of 1° × 1°. The twentieth century (1850–2014) historical and twenty-first century (2015–2100) future SSP scenarios (Table S2) were used to define historical and future temperature trends, respectively. SAT trend was influenced by different forcings. In the present study, we used the CMIP6 simulations of ALL, AER, NAT, GHG, and SOL which represent all, anthropogenic aerosol only, natural only, GHG only, and solar irradiance only forcings, respectively. A robust regression technique was used for the trend estimation, which could detect the effects of outliers and end points^36^. The low SSP1–2.6 and SSP2–4.5 scenarios and high SSP5–8.5 scenarios were used for the future projections (2015–2100).

Initially, the monthly anomalies of the historical (considered land data and discarded ocean data) datasets (ALL, AER, NAT, GHG, and SOL) and future scenarios (SSP1–2.6, SSP2–4.5, and SSP5–8.5) were collected from CMIP6. The simulations driven with ALL from 21 models, had 91 runs. The simulations driven by the other forcings of AER, NAT, GHG, and SOL included 10 (46), 10 (48), 11 (46), and 4 (22) models (runs), respectively (see Table 1). Similarly, the future projections driven by SSP1–2.6, SSP2–4.5, and SSP5–8.5 included an equal number of 8 (37) models (runs), respectively (see Table S2). The 1° × 1° gridded data were averaged for their respective models. Then, if the grids did not contain any observations, the corresponding grids of the models were masked out to match the observations. Initially, we averaged all the ensemble members (runs) for each model, and the MME mean was estimated as the equally weighted arithmetic average of all the individual model ensemble means. Subsequently, monthly anomalies were calculated for each grid (1° × 1°), and then annual (°C per year) and decadal tendencies (°C per decade) were evaluated relative to an 1850–2014 baseline. Finally, regional mean series were estimated based on the available grid values, weighted by the cosine of the latitude at the center of each grid box area, for each EA country, as well as the entire EA region.

### Regularized optimal fingerprinting (ROF) method

Detection and attribution analysis is a versatile tool that can be used to identify the drivers of climate change^25,37–40^. Several researchers have used this method to examine temperature increases from regional to global scales, with anthropogenic forcing being identified as the dominant factor in most cases^25,34,35,41^. Detection and attribution analyses have been performed using the Coupled Model Intercomparison Project Phase 3 (CMIP3)^42^, Phase 5 (CMIP5)^14,40,41^, and the new Phase 6 (CMIP6) model simulations^12,38^ to assess the relative contributions of various external forcings to climate change. Xu et al.^14^ used an optimal detection technique to identify distinct GHG- and ANT-associated temperature changes in China between 1961 and 2005; Yin et al.^43^ used multimodel CMIP5 simulations for the period 1958–2012 to detect ANT forcing of both extreme cold and warm temperatures in China; and Paik et al.^12^ reported that anthropogenic GHG emissions are the major contributor to extreme precipitation events across a range of climatic settings. As an attribution technique, the optimal fingerprinting method detects both anthropogenic and natural forcings (referred to as ‘ALL’ forcing) and ANT forcing^37,39^. Applying this approach to China, Wang et al.^25^ showed that warming in western China can be attributed to anthropogenic forcing, and also projected the future warming trends of this region. Yin et al.^43^ detected human influences on the intensity of extreme temperature changes in China, as well as at regional scales in eastern and western China, from 1958 to 2012. Lu et al.^41^ found anthropogenic influences on the frequency of daily temperature extremes in China using detection and attribution analysis. Furthermore, model‐simulated responses were consistent with observations of the daytime extremes. Many researchers have described anthropogenic influences on changes in temperature and precipitation over China^44–50^.

ROF was used for detection and attribution analysis based on the different forcings^40^. This approach can assess the contribution of external forcings based on the scaling factors in a linear regression model^37,40^. ROF is similar to the method of Allen and Stott^38^, except that a regularized covariance matrix is used for the optimization and estimation of the null distribution, which is used for the residual consistency test. ROF is based on the space–time evolution of SAT trends and is more accurate than the classical optimal detection method^40,51^, overcoming the limitation of empirical orthogonal function truncations. This method generates a full-rank measure of the covariance matrix of internal variability, which does not require empirical orthogonal function truncation^40,52^. Furthermore, it assumes that the climate response and noise signals are linearly additive, which means that the observed changes are the sum of climate response (externally forced change) and internally generated noise^3,51^. In ROF, the observations (Y) are regressed onto the MME mean signal patterns (X) using the total least squares method^40^, such that Y = Xβ + ε, where X^∗^  = X + ν. Here, ε is the noise from the internal variability in the observations, β contains the scaling factors that alter the magnitude of X, and X^∗^ is the matrix of signal patterns obtained from models, which are composed of a true but unknown matrix of signal patterns X plus noise from internal climate variability ν that remains in X^∗^ after multi-model averaging. The internal variability (ε) was estimated from the model simulations. We used pre-industrial control (CTL) simulations of 21 models (see Table S2) to estimate internal climate variability; this reduces the sampling uncertainty in covariance estimations^40^. These CTL simulations were divided into 283 non-overlapping segments that were each 110 years long (the segment length was equal to the 1905–2014 period used for the detection analysis). The CTL simulations were divided into two sets of equal size, one was used for optimization and to derive the best estimates, and the other was used to calculate the 5–95% confidence intervals of the scaling factors and also to carry out the residual consistency test^4,37,40^. The residual consistency test uses a nonparametric estimation of the null distribution through Monte-Carlo simulations to determine whether the noise estimate (ε) is consistent with the simulated internal variability^39,52^. The influence of the different forcings was detected based on β. A 90% confidence interval of β greater than zero implies that a corresponding influence of external forcing has been detected. If the 90% range of β is above zero and it includes unity, this indicates that the observed change is consistent with the model simulations. If the 90% interval of β is greater than unity, this implies that the observed changes are underestimated by the model simulations. For further details regarding the ROF method, refer to Ribes et al.^40^ and Ribes and Terray^39^.

For detection and attribution, we used the MME means of the available simulations for ALL, GHG, ANT, and NAT, which largely smoothed the uncorrelated internal variations^51^. ANT was estimated by subtracting NAT from ALL (ANT = ALL − NAT) and CRU data used as the observational data. We conducted one-signal, two-signal, and three-signal analyses over the last hundred years (1905–2014) for each EA country and EA overall. Detection and attribution are most effective at small data dimensions, allowing better estimation of climate response and, thus, are usually conducted within shorter dimensions^14^. Therefore, we converted the annual data sets into non-overlapping ten-year means for the 1905–2014 period, which produced 11 data values (1905–1914, 1915–1924, 1925–1934, … and 2005–2014). The use of 10-year temporal means reduced the time dimension, as well as the variability of the observations and noise in the climate signals^25,51^. In the one-signal analysis (Eq. 1), the observations were regressed onto MME mean responses of ALL and ANT fingerprints separately to detect their relative influence on the observed change. In the two-signal analysis (Eq. 2), the observations were simultaneously regressed onto the MME mean responses of ANT and NAT fingerprints to determine their separate contributions. In the three-signal analysis (Eq. 4), the observations were regressed onto the MME mean response patterns from the GHG, ANTnoGHG (i.e., ANT–GHG), and NAT simulations simultaneously to clearly detect and isolate the GHG forcing effect. In the other three-signal analysis (Eq. 8), the observations were regressed onto ALL, AER, and NAT (Eq. 6) and the regression coefficients were then transformed to obtain the GHG, AER, and NAT^53^. GHG, AER, and NAT scaling factors are calculated by decomposing Eq. (7) and substituting into Eq. (8), where β_GHG_ = β_1_, β_AER_ = β_1_ + β_2_, β_NAT_ = β_1_ + β_3_, and the resultant is illustrated in Eq. (9).

1 $${\text{Y}}_{{{\text{OBS}}}} = {\upbeta }_{{\text{M}}} {\text{X}}_{{\text{M}}} + {\upvarepsilon }$$

2 $${\text{Y}}_{{{\text{OBS}}}} = {\upbeta }_{{{\text{ANT}}}} {\text{X}}_{{{\text{ANT}}}} + {\upbeta }_{{{\text{NAT}}}} {\text{X}}_{{{\text{NAT}}}} + {\upvarepsilon }$$

3 $${\text{X}}_{{{\text{ALL}}}} = {\text{X}}_{{{\text{ANT}}}} + {\text{X}}_{{{\text{NAT}}}}$$

4 $${\text{Y}}_{{{\text{OBS}}}} = {\upbeta }_{{{\text{GHG}}}} {\text{X}}_{{{\text{GHG}}}} + {\upbeta }_{{{\text{ANTnoGHG}}}} {\text{X}}_{{{\text{ANTnoGHG}}}} + {\upbeta }_{{{\text{NAT}}}} {\text{X}}_{{{\text{NAT}}}} + {\upvarepsilon }$$

5 $${\text{X}}_{{{\text{ALL}}}} = {\text{X}}_{{{\text{GHG}}}} + {\text{X}}_{{{\text{ANTnoGHG}}}} + {\text{X}}_{{{\text{NAT}}}}$$

6 $${\text{Y}}_{{{\text{OBS}}}} = {\upbeta }_{1} {\text{X}}_{{{\text{ALL}}}} + {\upbeta }_{2} {\text{X}}_{{{\text{AER}}}} + {\upbeta }_{3} {\text{X}}_{{{\text{NAT}}}} + {\upvarepsilon }$$

7 $${\text{X}}_{{{\text{ALL}}}} = {\text{X}}_{{{\text{GHG}}}} + {\text{X}}_{{{\text{AER}}}} + {\text{X}}_{{{\text{NAT}}}}$$

8 $${\text{Y}}_{{{\text{OBS}}}} = {\upbeta }_{{{\text{GHG}}}} {\text{X}}_{{{\text{GHG}}}} + {\upbeta }_{{{\text{AER}}}} {\text{X}}_{{{\text{AER}}}} + {\upbeta }_{{{\text{NAT}}}} {\text{X}}_{{{\text{NAT}}}} + {\upvarepsilon }$$

9 $${\text{Y}}_{{{\text{OBS}}}} = {\upbeta }_{1} {\text{X}}_{{{\text{GHG}}}} + \left( {{\upbeta }_{1} + {\upbeta }_{2} } \right){\text{X}}_{{{\text{AER}}}} + \left( {{\upbeta }_{1} + {\upbeta }_{3} } \right){\text{X}}_{{{\text{NAT}}}} + {\upvarepsilon }$$

where Y_OBS_ represents the observations; X indicates the model simulations; M is the fingerprints of ALL, GHG, ANT, ANTnoGHG, and NAT; β is the unknown regression coefficient or scaling factor; ε is the regression residual term, which represents the noise due to internal variability; X_ALL_, X_GHG_, X_ANT_, X_ANTnoGHG_, and X_NAT_ are the model simulation responses to the ALL, GHG, ANT, ANTnoGHG, and NAT forcings, respectively; and β_GHG_, β_ANT_, β_ANTnoGHG_, and β_NAT_ are scaling factors corresponding to the GHG, ANT, ANTnoGHG, and NAT forcings, respectively.

The historical simulations show mostly stable values in the first few decades, followed by increasing/decreasing patterns. Due to the uncertainty in the historical record, it is difficult to maintain consistency between historical and future scenarios; additionally, different scenarios do not include the same set of human activities that cause emissions. As a result, it is critical to provide smooth and accurate future projections. Therefore, future temperature projections were assessed using a common historical reference period. To ensure a smooth transition from historical to future projections, observation-constrained future projections were calculated by subtracting each future year from the average of a historical reference period (recent past years 1995–2014)^32,33^. The future values were then adjusted (underestimation/overestimation) by multiplying by the best estimate of the scaling factor calculated in the detection analysis (Fig. 6).

## Supplementary Information

Supplementary Information.

## Data Availability

The datasets used in the present study are: HadCRUT4, available at https://crudata.uea.ac.uk/cru/data/temperature/; CRU, available at https://crudata.uea.ac.uk/cru/data/hrg/; and CMIP6, available at https://esgf-node.llnl.gov/projects/cmip6/.

## Code availability

The code for the regularized optimal fingerprinting method used for detection and attribution analysis is available at http://www.umr-cnrm.fr/spip.php?article23&lang=en.
