# Supplementary material for: Anthropogenic influence of temperature changes across East Asia using CMIP6 simulations
Source: Sci Rep. 2022 Jul 13;12:11896. doi: 10.1038/s41598-022-16110-9 (PMC9279311; doi:10.1038/s41598-022-16110-9)
Supplement: Supplementary file 1 — Supplementary Information. [file 41598_2022_16110_MOESM1_ESM.docx]

**Anthropogenic influence of temperature changes across East Asia using CMIP6 simulations**

Shaik Allabakash^1,2^, Sanghun Lim^1*^

^1^Korea Institute of Civil Engineering and Building Technology, Republic of Korea

^2^Météo-France, Centre de Météorologie Radar, Toulouse, France

*Corresponding author: Sanghun Lim

283, Korea Institute of Civil Engineering and Building Technology, Goyang-daero, Ilsanseo-gu, Goyang-si, Gyeonggi-do 10223, Republic of Korea.

Email: [[slim@kict.re.kr](mailto:slim@kict.re.kr)](mailto:email@address.edu))

Figure S1. Spatial surface air temperature trends for 1901-2014 (a) CRU, (b) Multi-model ensemble mean simulations response to ALL forcing of CMIP6, and (c) Spatial surface air temperature difference between CRU and ALL forcing of CMIP6. All figures were generated using licensed MATLAB (release R2017b available at https://in.mathworks.com/products/matlab.html).

Figure S2. Spatial distribution of trends in annual mean surface air temperature responses to different forcings (°C/decade) for the historical period 1971–2014. (a) ALL, (b) anthropogenic aerosol (AER), (c) natural (NAT), (d) greenhouse gas (GHG), and (e) solar irradiance (SOL) forcings. The trends are estimated based on robust regression analysis. All figures were generated using licensed MATLAB (release R2017b available at https://in.mathworks.com/products/matlab.html).

Figure S3. Temporal variation of annual mean temperature anomaly responses to natural (NAT) forcing averaged across East Asia and individual countries from observations (CRU and HadCRUT4) and multi-model mean simulations (CMIP6) for the period 1850–2014 (CRU: 1901–2018, HadCRUT4: 1850–2019). Shaded bands are multi-model ranges.

Figure S4. As for Figure S3 but showing temperature anomaly responses to solar (SOL) forcing.

Figure S5. Temporal variations in annual SAT anomalies (relative to 1995–2014) during the period 1850–2100. Shaded bands are the multi-model ranges. Future projections are based on the multi-model ensemble means under SSP1–2.6 (gold line), SSP2–4.5 (green line), and SSP5–8.5 (magenta line). The dashed lines indicate best-estimate observation-constrained future temperature projections for (a) East Asia, (b) China, (c) Mongolia, (d) Japan, and (e) Korea. The future projections (solid lines) obtained by taking the difference of each future year with respect to the average from the ALL forcing historical period (1995-2014) and are multiplied with the best estimate of the scaling factors of ALL and obtained the adjusted/corrected projections.

Figure S6. Temporal variations in annual SAT anomalies (relative to 1995–2014) during the period 1850–2100. Shaded bands are the multi-model ranges. Future projections are based on the multi-model ensemble means under SSP1–2.6 (gold line), SSP2–4.5 (green line), and SSP5–8.5 (magenta line). The dashed lines indicate best-estimate observation-constrained future temperature projections for (a) East Asia, (b) China, (c) Mongolia, (d) Japan, and (e) Korea. The future projections (solid lines) obtained by taking the difference of each future year with respect to the average from the GHG forcing historical period (1995-2014) and are multiplied with the best estimate of the scaling factors of GHG (figure 6 and obtained the adjusted/corrected projections.

**Tables**

Table S1. Annual mean surface air temperature trend (°C/decade) responses to different forcings in each East Asian country and the entire EA region for the period 1850–2014 (P1) and 1971–2014 (P2, after third industrial revolution, shown in parenthesis). ALL = all, AER = anthropogenic aerosol, NAT = natural, GHG = greenhouse gas, and SOL = solar irradiance.

Table S2. Models used in the study for the future projections (Y = ‘yes’ and ‘N’ = no). The numbers in parenthesis represent the ensemble sizes of the corresponding models

Table S3. Best estimates and 90% confidence intervals (shown in parenthesis) of future scenarios (SSP1-2.6, SSP2-4.5, and SSP5-8.5) in East Asian countries estimated based on best estimate of ALL forcing simulations of one-signal analysis.

Table S4. Best estimates and 90% confidence intervals (shown in parenthesis) of future scenarios (SSP1-2.6, SSP2-4.5, and SSP5-8.5) in East Asian countries estimated based on best estimate of GHG forcing simulations of three-signal analysis (see Figure 6d and Table 2).


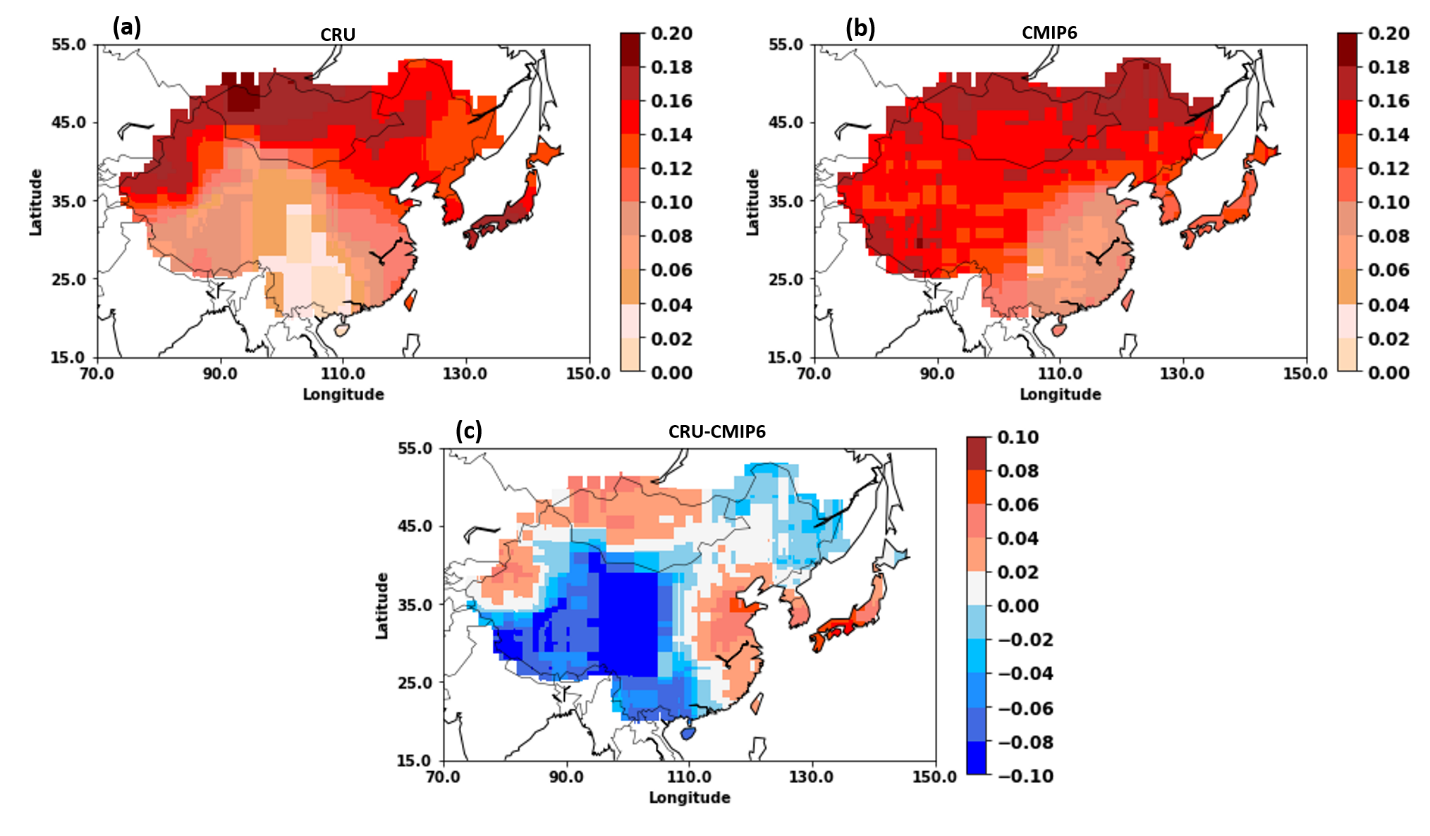


Figure S1. Spatial surface air temperature trends for 1901-2014 (a) CRU, (b) Multi-model ensemble mean simulations response to ALL forcing of CMIP6, and (c) Spatial surface air temperature difference between CRU and ALL forcing of CMIP6. All figures were generated using licensed MATLAB (release R2017b available at https://in.mathworks.com/products/matlab.html).


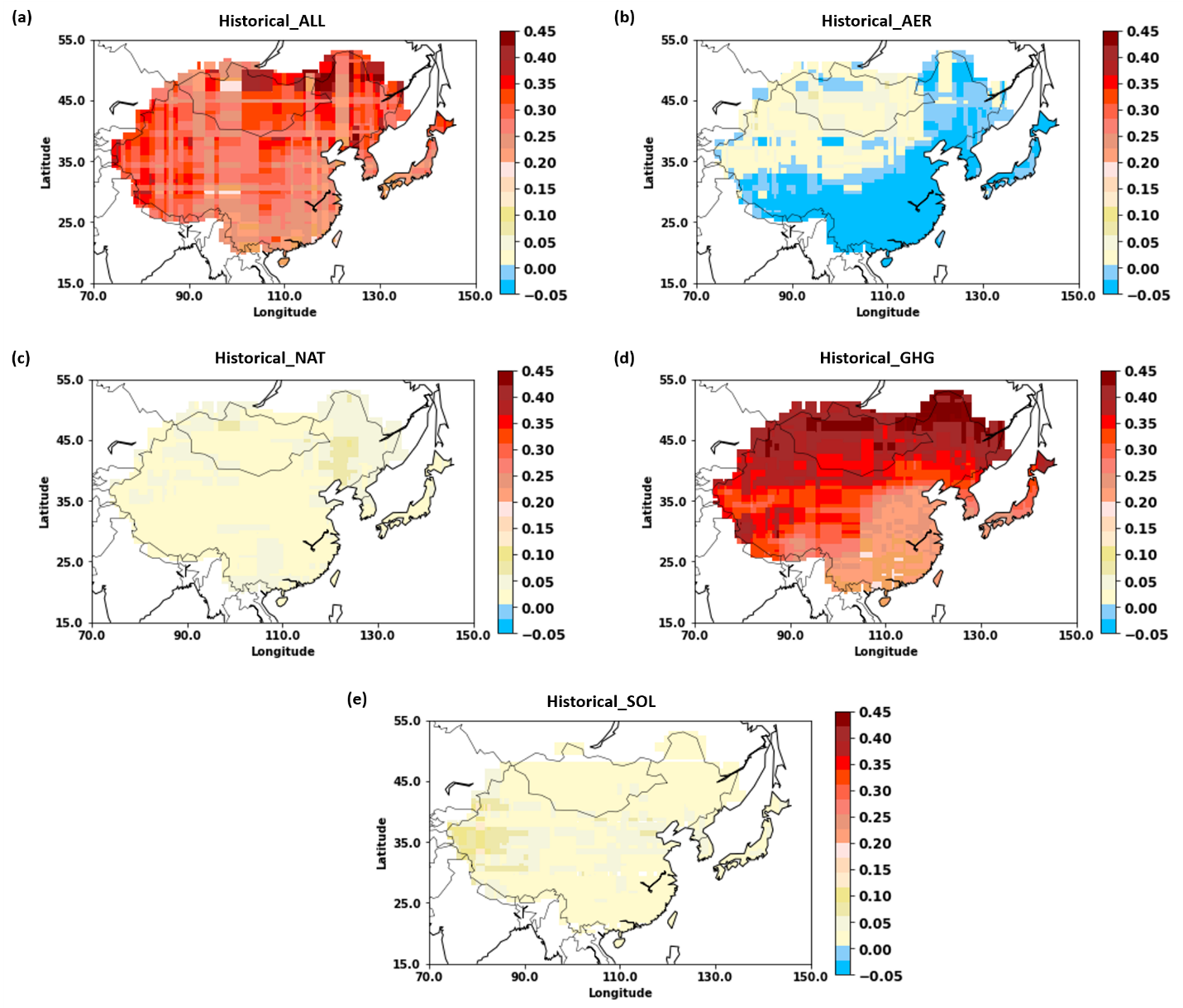


Figure S2 Spatial distribution of trends in annual mean surface air temperature responses to different forcings (°C/decade) for the historical period 1971–2014. (a) ALL, (b) anthropogenic aerosol (AER), (c) natural (NAT), (d) greenhouse gas (GHG), and (e) solar irradiance (SOL) forcings. The trends are estimated based on robust regression analysis. All figures were generated using licensed MATLAB (release R2017b available at https://in.mathworks.com/products/matlab.html).


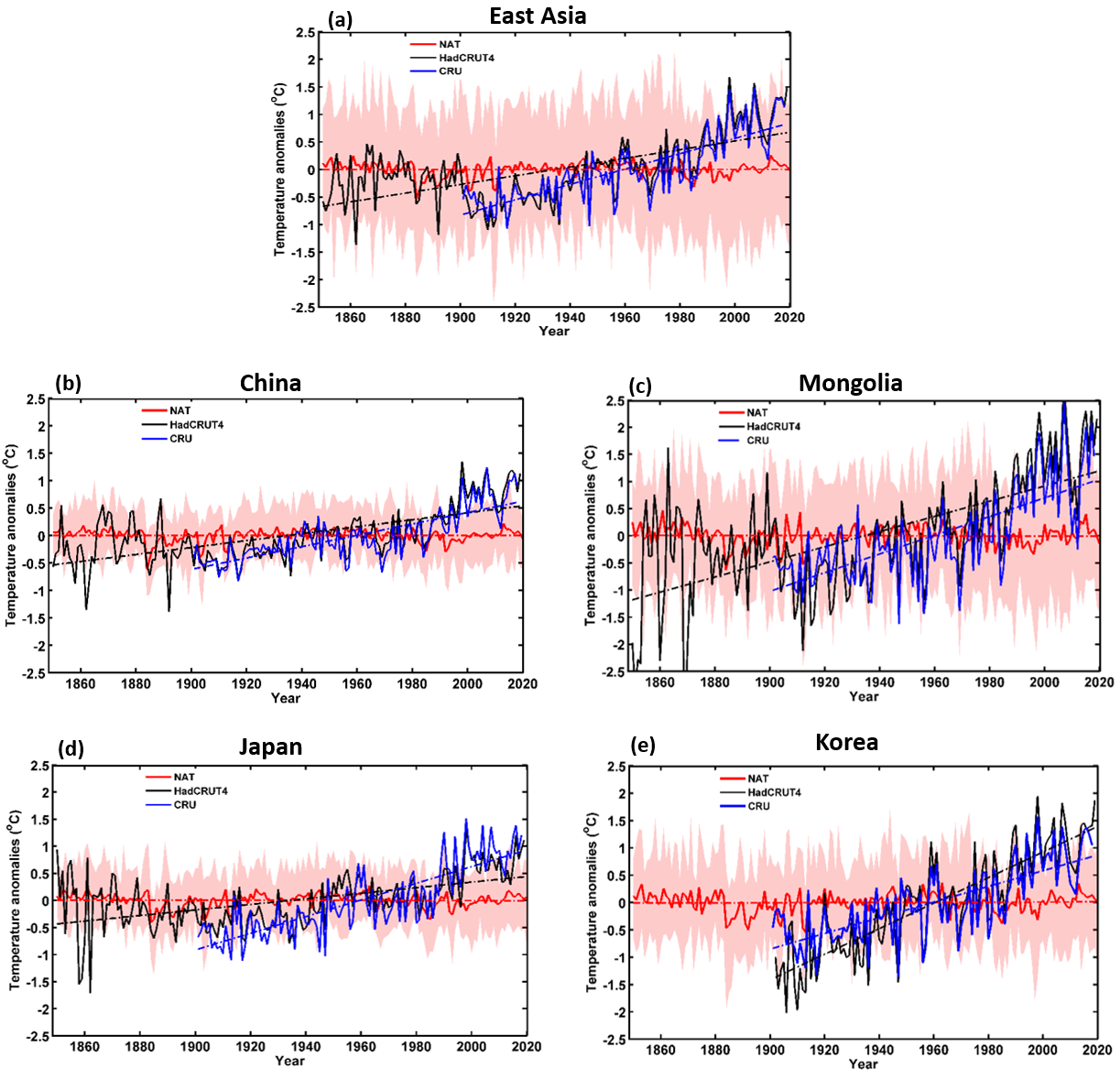


Figure S3. Temporal variation of annual mean temperature anomaly responses to natural (NAT) forcing averaged across East Asia and individual countries from observations (CRU and HadCRUT4) and multi-model mean simulations (CMIP6) for the period 1850–2014 (CRU: 1901–2018, HadCRUT4: 1850–2019). Shaded bands are multi-model ranges.


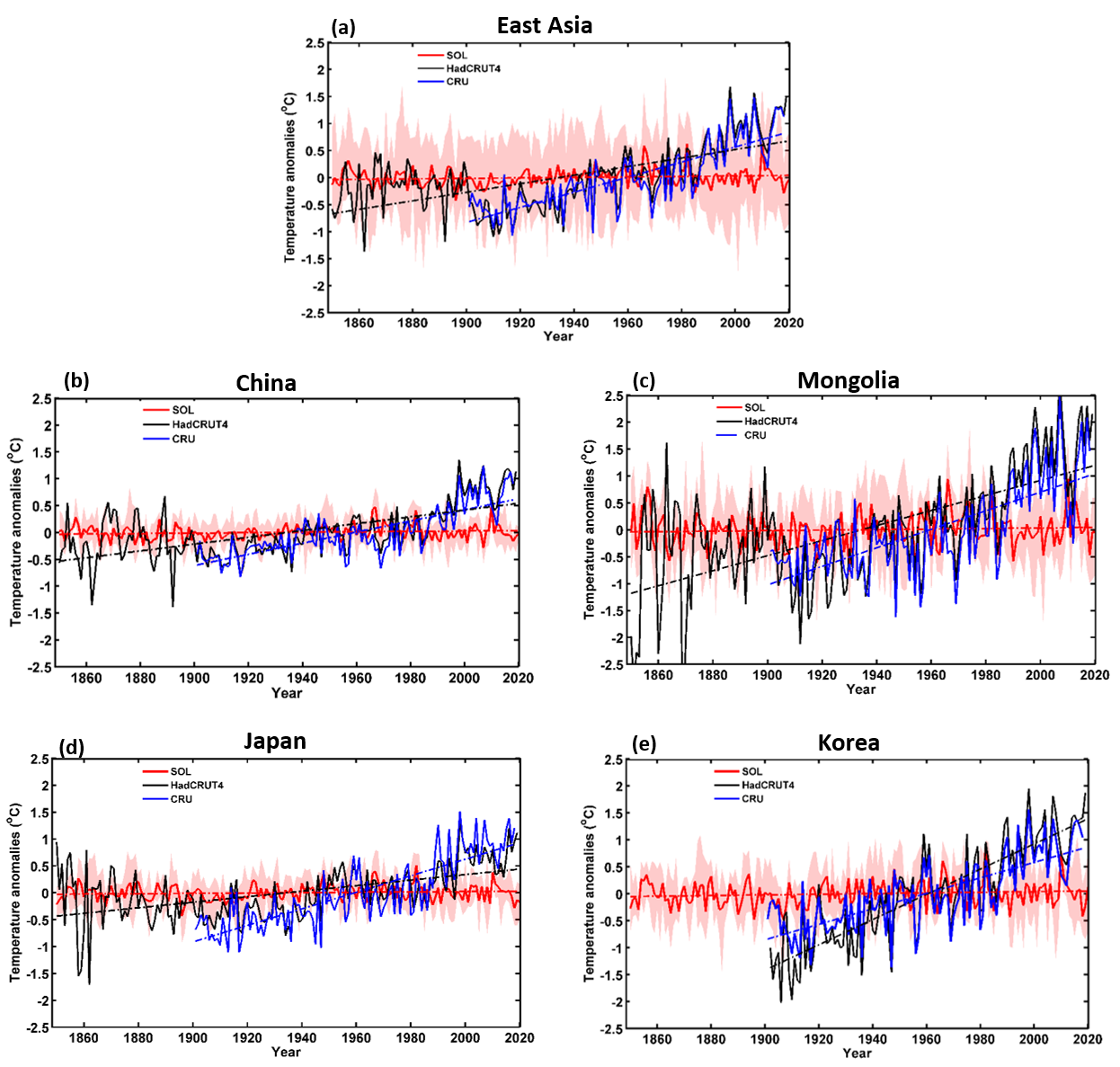


Figure S4. As for Figure S3 but showing temperature anomaly responses to solar (SOL) forcing.


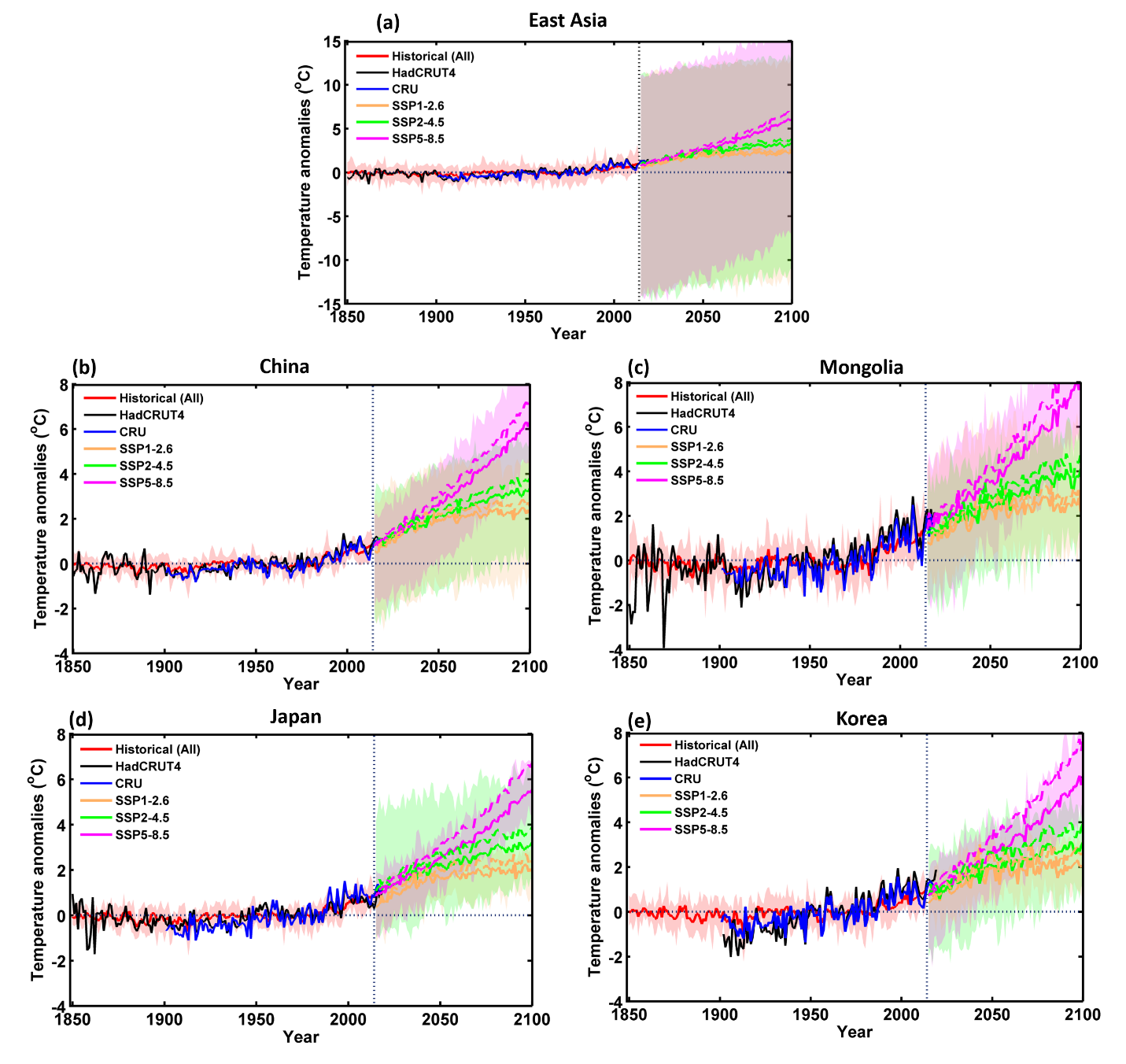


Figure S5 Temporal variations in annual SAT anomalies (relative to 1995–2014) during the period 1850–2100. Shaded bands are the multi-model ranges. Future projections are based on the multi-model ensemble means under SSP1–2.6 (gold line), SSP2–4.5 (green line), and SSP5–8.5 (magenta line). The dashed lines indicate best-estimate observation-constrained future temperature projections for (a) East Asia, (b) China, (c) Mongolia, (d) Japan, and (e) Korea. The future projections obtained by taking the difference of each future year with respect to the average from the ALL forcing historical period (1995-2014) and are multiplied with the best estimate of the scaling factors of ALL and obtained the adjusted/corrected projections.


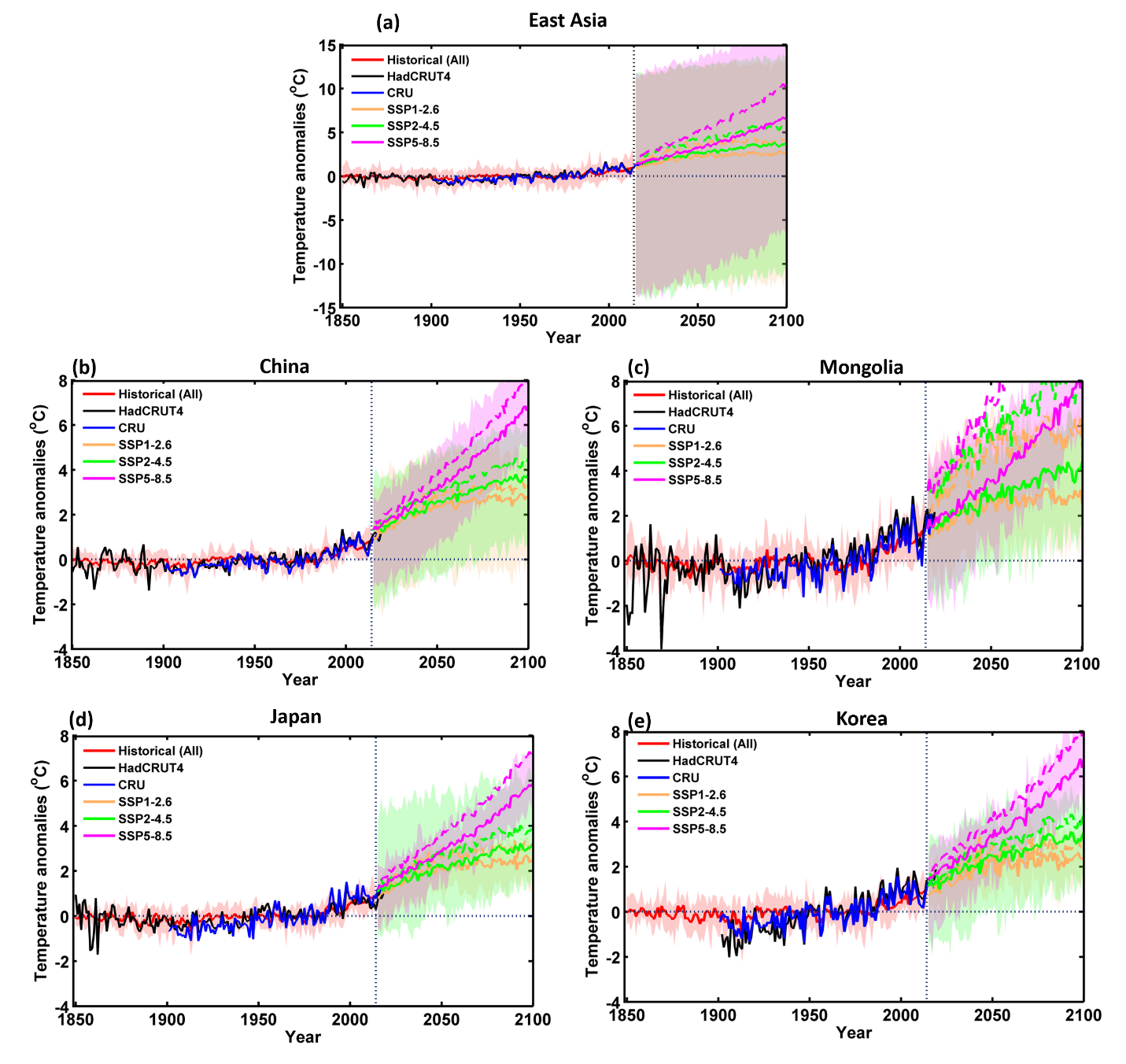


Figure S6 Temporal variations in annual SAT anomalies (relative to 1995–2014) during the period 1850–2100. Shaded bands are the multi-model ranges. Future projections are based on the multi-model ensemble means under SSP1–2.6 (gold line), SSP2–4.5 (green line), and SSP5–8.5 (magenta line). The dashed lines indicate best-estimate observation-constrained future temperature projections for (a) East Asia, (b) China, (c) Mongolia, (d) Japan, and (e) Korea. The future projections obtained by taking the difference of each future year with respect to the average from the GHG forcing historical period (1995-2014) and are multiplied with the best estimate of the scaling factors of GHG (figure 6) and obtained the adjusted/corrected projections.

**Tables**

Table S1. Annual mean surface air temperature trend (°C/decade) responses to different forcings in each East Asian country and the entire EA region for the period 1850–2014 (P1) and 1971–2014 (P2, after third industrial revolution, shown in parenthesis). ALL = all, AER = anthropogenic aerosol, NAT = natural, GHG = greenhouse gas, and SOL = solar irradiance

| 1850–2014  (1971–2014) | **East Asia** | **China** | **Mongolia** | **Japan** | **Korea** |
| --- | --- | --- | --- | --- | --- |
| **ALL** | 0.031  (0.255) | 0.027  (0.223) | 0.049  (0.379) | 0.026  (0.25) | 0.029  (0.266) |
| **AER** | −0.076  (−0.035) | −0.081  (−0.058) | −0.086  (0.010) | −0.068  (−0.036) | -0.078  (-0.041) |
| **NAT** | 0.0085  (0.019) | −0.0012  (0.013) | −0.002  (0.020) | −0.002  (0.015) | -0.002  (0.036) |
| **GHG** | 0.082  (0.268) | 0.090  (0.27) | 0.1106  (0.328) | 0.077  (0.240) | 0.087  (0.278) |
| **SOL** | 0.005  (0.008) | 0.005  (−0.001) | 0.006  (0.007) | 0.005  (0.009) | 0.005  (0.029) |

Table S2. Models used in the study for the future projections (Y = ‘yes’ and ‘N’ = no). The numbers in parenthesis represent the ensemble sizes of the corresponding models

| **Model name** | **Historical (1850-2014)** | | | | **Future projection (2014–2100)** | | | **Control simulations** |
| --- | --- | --- | --- | --- | --- | --- | --- | --- |
|  | ALL | AER | NAT | GHG | SSP1-2.6 | SSP2-4.5 | SSP5-8.5 | CTL |
| MRI-ESM2-0 | Y (3) | Y (3) | Y (3) | Y (3) | Y (3) | Y (3) | Y (3) | Y (8) |
| CIESM | N | N | N | N | N | N | N | Y (20) |
| CAMS-CSM1-0 | N | N | N | N | N | N | N | Y (16) |
| INM-CM4-8 | N | N | N | N | N | N | N | Y (12) |
| FIO-ESM-2-0 | N | N | N | N | N | N | N | Y (15) |
| CMCC-CM2 | N | N | N | N | N | N | N | Y (12) |
| HadGEM3- GC31-MM | N | N | N | N | N | N | Y (4) | Y (16) |
| MIROC6 | Y (2) | Y (2) | Y (2) | Y (2) | Y (2) | Y(2) | Y (2) | N |
| GISS-E2-1-G | Y (5) | Y (5) | Y (5) | Y (5) | Y (5) | Y (5) | Y (5) | Y (12) |
| CNRM-CM6-1 | Y (5) | Y (5) | Y (5) | Y (5) | Y (5) | Y (5) | Y (5) | Y (20) |
| IPSL-CM6A-LR | Y (4) | Y (4) | Y (4) | Y (4) | Y (4) | Y (4) | Y (4) | Y (6) |
| CNRM-ESM2-1 | N | N | N | N | N | N | N | Y (16) |
| ACCESS- ESM1-5 | Y (6) | Y (6) | Y (6) | Y (6) | Y (6) | Y (6) | Y (6) | N |
| HadGEM3- GC31-LL | Y (4) | Y (4) | Y (4) | Y (4) | Y (4) | Y (4) | Y (4) | Y (12) |
| UKESM1-0-LL | N | N | N | N | N | N | N | Y (10) |
| CanESM5 | Y (4) | Y (4) | Y (4) | Y (4) | Y (4) | Y (4) | Y (4) | N |
| CMCC-ESM2 | N | N | N | N | N | N | N | Y (16) |
| **MPI-ESM1-2-LR** | N | N | N | N | N | N | N | Y (18) |
| CESM2 | N | N | N | N | N | N | N | Y (20) |
| **FGOALS-f3-L** | N | N | N | N | N | N | N | Y (15) |
| NESM3 | N | N | N | N | N | N | N | Y (8) |
| **KACE-1-0-G** | N | N | N | N | N | N | N | Y (8) |
| **Total** | 8 (37) | 8 (37) | 8 (37) | 8 (37) | 8 (37) | 8 (37) | 8 (37)) | 21  (283) |

Table S3. Best estimates and 90% confidence intervals (shown in parenthesis) of future scenarios (SSP1-2.6, SSP2-4.5, and SSP5-8.5) in East Asian countries estimated based on best estimate of ALL forcing simulations of one-signal analysis.

| **Future scenarios** | **Year** | **East Asia**  **(90%)** | **China**  **(90%)** | **Mongolia**  **(90%)** | **Japan**  **(90%)** | **Korea**  **(90%)** |
| --- | --- | --- | --- | --- | --- | --- |
| **SSP1-2.6** | 2041-2060 | 2.15  (0.15-4.21) | 2.21  (0.96-3.47) | 2.65  (1.38-3.93) | 2.09  (1.60-2.57) | 2.24  (1.47-3.02) |
|  | 2061-2080 | 2.35  (0.39-4.42) | 2.47  (1.16-3.77) | 2.87  (1.58-4.16) | 2.28  (1.72-2.85) | 2.35  (1.52-3.18) |
|  | 2081-2100 | 2.48  (0.44-4.55) | 2.60  (1.29-3.9) | 2.96  (1.69-4.24) | 2.44  (1.91-2.97) | 2.51  (1.64-3.38) |
| **SSP2-4.5** | 2041-2060 | 2.43  (0.96-3.93) | 2.39  (1.38-3.40) | 2.93  (1.83-4.03) | 2.62  (1.80-3.44) | 2.31  (1.42-3.21) |
|  | 2061-2080 | 3.0  (1.51-4.49) | 3.02  (2.02-4.03) | 3.67  (2.63-4.72) | 3.16  (2.36-3.97) | 2.87  (2.01-3.73) |
|  | 2081-2100 | 3.51  (2.03-4.98) | 3.56  (2.61-4.52) | 4.31  (3.41-5.20) | 3.62  (2.88-4.37) | 3.39  (2.60-4.17) |
| **SSP5-8.5** | 2041-2060 | 2.97  (1.49-4.46) | 3.02  (2.24-3.79) | 4.12  (3.09-5.16) | 3.08  (2.73-3.42) | 3.17  (2.64-3.69) |
|  | 2061-2080 | 4.39  (2.95-5.83) | 4.52  (3.76-5.28) | 5.99  (5.04-694) | 4.31  (3.99-4.62) | 4.59  (4.1-5.08) |
|  | 2081-2100 | 6.11  (4.71-7.50) | 6.28  (5.48-7.08) | 8.14  (7.37-8.91) | 5.93  (5.55-6.31) | 6.35  (5.79-6.91) |

Table S4. Best estimates and 90% confidence intervals (shown in parenthesis) of future scenarios (SSP1-2.6, SSP2-4.5, and SSP5-8.5) in East Asian countries estimated based on best estimate of GHG forcing simulations of three-signal analysis (see Figure 6d and Table 2).

| **Future scenarios** | **Year** | **East Asia**  **(90%)** | **China**  **(90%)** | **Mongolia**  **(90%)** | **Japan**  **(90%)** | **Korea**  **(90%)** |
| --- | --- | --- | --- | --- | --- | --- |
| **SSP1-2.6** | 2041-2060 | 3.21  (0.60-5.82) | 2.73  (1.42-4.04) | 5.03  (2.84-7.22) | 2.58  (2.08-3.08) | 2.62  (1.84-3.40) |
|  | 2061-2080 | 3.46  (0.84-6.08) | 3.00  (1.65-4.35) | 5.40  (3.19-7.61) | 2.78  (2.20-3.37) | 2.73  (1.89-3.56) |
|  | 2081-2100 | 3.63  (1.01-6.25) | 3.13  (1.78-4.48) | 5.57  (3.38-7.75) | 2.94  (2.40-3.49) | 2.89  (2.01-3.76) |
| **SSP2-4.5** | 2041-2060 | 3.85  (1.93-5.76) | 2.92  (1.87-3.97) | 5.28  (3.34-7.97) | 2.75  (1.90-3.59) | 2.94  (2.03-3.84) |
|  | 2061-2080 | 4.58  (2.68-6.47) | 3.57  (2.53-4.62) | 7.06  (4.62-9.50) | 3.31  (2.48-4.14) | 3.50  (2.63-4.37) |
|  | 2081-2100 | 5.22  (3.34-7.09) | 4.13  (3.14-5.12) | 8.96  (7.26-10.42) | 3.78  (3.01-4.55) | 4.02  (3.23-4.81) |
| **SSP5-8.5** | 2041-2060 | 4.72  (2.83-6.60) | 3.78  (2.98-4.58) | 6.73  (5.01-8.35) | 3.53  (3.18-3.89) | 4.11  (3.58-4.63) |
|  | 2061-2080 | 6.52  (4.69-8.35) | 5.34  (4.55-6.12) | 8.89  (8.01-10.67) | 4.80  (4.48-5.13) | 5.54  (5.05-6.04) |
|  | 2081-2100 | 8.70  (6.93-10.47) | 7.16  (6.33-799) | 14.73  (13.96-16.92) | 6.48  (6.09-6.87) | 7.32  (6.75-7.88) |
